# Inference of future bog succession trajectory from spatial chronosequence of changing aapa mires

**DOI:** 10.1002/ece3.9988

**Published:** 2023-04-18

**Authors:** Tiina H. M. Kolari, Teemu Tahvanainen

**Affiliations:** ^1^ Department of Environmental and Biological Sciences University of Eastern Finland P.O. Box 111 Joensuu FI‐80101 Finland

**Keywords:** fen‐bog transition, future ecosystems, hydroseral development, patterned fen, peatland, plant population and community dynamics, spatial chronosequence, *Sphagnum*, succession, vegetation change

## Abstract

Climate change‐driven vegetation changes can alter the ecosystem functions of northern peatlands. Several case studies have documented fen‐to‐bog transition (FBT) over recent decades, which can have major implications, as increased bog growth would likely cause cooling feedback. However, studies beyond individual cases are missing to infer if a common trajectory or many alternatives of FBT are in progress. We explored plant community and hydrology patterns during FBT of 23 boreal aapa mire complexes in Finland. We focused on mires where comparisons of historical (1940–1970) and new (2017–2019) aerial photographs indicated an expansion of *Sphagnum*‐dominated zones. Vegetation plot and water chemistry data were collected from string‐flark fens, transition zones with indications of *Sphagnum* increase, and bog zones; thus, in a chronosequence with a decadal time span. We ask, is there a common trajectory or many alternatives of FBT in progress, and what are the main characteristics (species and traits) of transitional plant communities? We found a pattern of fen‐bog transitions via an increase in *Sphagnum* sect. *Cuspidata* (mainly *S*. *majus* and *S*. *balticum*), indicating a consistently high water table. Indicators only of transitional communities were scarce (*Sphagnum lindbergii*), but FBT had apparently facilitated shallow‐rooted aerenchymatous vascular plants, especially *Scheuchzeria palustris*. Water pH consistently reflected the chronosequence with averages of 4.2, 3.9, and 3.8, from fen to transition and bog zones. Due to weak minerotrophy of string‐flark fens, species richness increased towards bogs, but succession led to reduced beta diversity and homogenization among bog sites. Decadal chronosequence suggested a future fen‐bog transition through a wet phase, instead of a drying trend. Transitional poor‐fen vegetation was characterized by the abundance of *Sphagnum lindbergii*, *S*. *majus*, and *Scheuchzeria palustris*. *Sphagnum* mosses likely benefit from longer growing seasons and consistently wet and acidic conditions of aapa mires.

## Graphical abstract

We studied recent fen‐bog transitions and the characteristics of transitional plant communities in 23 boreal aapa mires in a spatial chronosequence with a decadal time span. We found a pattern of fen‐bog transitions via an increase in wet Sphagna (*Sphagnum lindbergii*, *S*. *majus*, and *S*. *balticum*) and *Scheuchzeria palustris*, highlighting the fact that under climate change, boreal fens may experience a shift to *Sphagnum*‐dominated bogs through a wet phase.

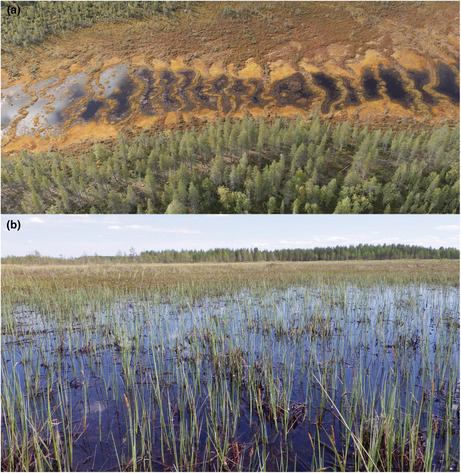

## 1 INTRODUCTION

Climate change and land use have a wide range of consequences on ecosystems. In high‐latitude mires (peat‐accumulating wetlands), rising temperatures and lengthening of the growing season enhance gross primary production and promote the growth of decay‐resistant peat mosses (*Sphagnum* spp.; Charman et al., 2013; Gallego‐Sala et al., 2018; Kolari et al., 2021; Loisel et al., 2012; Ma et al., 2022), which can lead to an ecosystem‐level change from sedge‐dominated fens to *Sphagnum*‐dominated bogs (Kolari et al., 2022; Loisel & Yu, 2013; Magnan et al., 2022; Tahvanainen, 2011). The fen‐bog transition (FBT) can alter the climate feedback of mires by increasing carbon sequestration (Granlund et al., 2022; Loisel & Yu, 2013) and by reducing methane emission (Juottonen et al., 2022; Zhang et al., 2021), while it poses threat to the biodiversity of fen habitats. Recently, many studies of high‐boreal and subarctic peatlands have indicated increased carbon sequestration and FBT with warming after the Little Ice Age (LIA) and during the 20th century (Granlund et al., 2022; Loisel & Yu, 2013; Magnan et al., 2018, 2022; Piilo et al., 2019; Primeau & Garneau, 2021; Robitaille et al., 2021). The FBT is well‐known from peat stratigraphies: *Sphagnum* peat in bogs is most often underlain by sedge peat of earlier fen phases, and the transition tends to be relatively short in duration (Hughes & Barber, 2003; Kuhry et al., 1993; Tuittila et al., 2013) and connected to a shift from high to low pH (Gorham & Janssens, 1992).

The general correspondence of the historical FBT phases in peat profiles with vegetation gradient among contemporary mires supports the approach to consider sequences from fen‐to‐bog sites as spatial chronosequences of the FBT. However, fens are highly variable in vegetation and water chemistry (Tahvanainen, 2004), and the FBT has alternative trajectories (Hughes & Barber, 2004). Thus, interpretation as chronosequence needs to be well tied to recognized processes and pace of changes (Walker et al., 2010). For example, one much‐used peatland chronosequence consists of sites along the land‐uplift coast in Finland, where the sequence of mires from primary paludification to fen, transition fen, and bog represents a 4000‐year chronosequence of bog development (Juottonen et al., 2022; Laine et al., 2021; Rehell & Virtanen, 2016; Tuittila et al., 2013). However, the connection to decadal timescale changes, relevant to the assessment of 21st‐century climate change impacts, is uncertain from long‐term peatland development studies of peat stratigraphies and chronosequences recognized so far.

**FIGURE 1:**
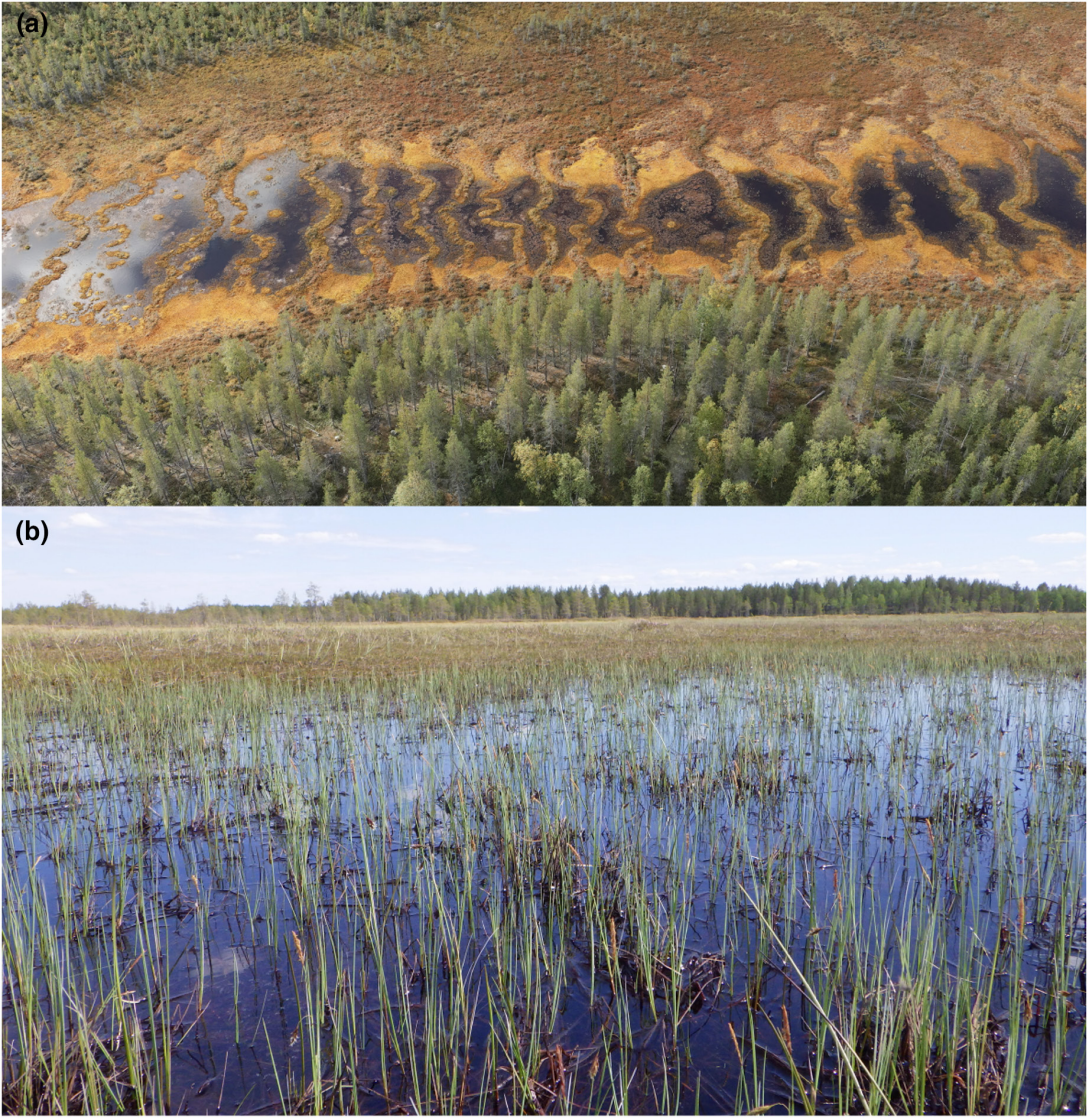
(a) An aapa mire complex, showing the characteristic string‐flark pattern in the central fen area and a surrounding bog area with hummocks and lawns. The margins between these main zones have fen to bog gradients that are informative of potential fen‐bog transitions (FBT) in the present climate conditions. (b) A close‐up image of a fen flark, with sparse and partly submerged vegetation dominated by *Carex limosa* and *C. rostrata*. Sources of images: Pasi Korpelainen (UAV image) and Teemu Tahvanainen (lower photograph).

Aapa mires comprise the most abundant mire complex type in northern Fennoscandia (Cajander, 1913; Laitinen et al., 2007; Ruuhijärvi, 1960). Aapa mires are also referred to as string‐flark mires, as they have characteristic patterning of hummock strings and hollows called “flarks” with sparse or submerged vegetation that form against the slope and water flow (Figure 1). In the broad sense, aapa mires are synonymous with patterned fens, and they are widespread in the boreal regions of North America (Garneau et al., 2018; Vitt et al., 2022; White & Payette, 2016) and Russia (Kutenkov et al., 2022; Yurkovskaya, 2012). Aapa mires are mire complexes with ombrotrophic bog vegetation in the margins, while central areas are characterized by string‐flark fen vegetation (Ruuhijärvi, 1960). The margins between the main zones in these mire complexes have fen‐to‐bog gradients that are informative of potential FBT in aapa mires. Several case studies have shown rapid lateral expansion of bog vegetation across these fen‐to‐bog gradients (Granlund et al., 2022; Kolari et al., 2022), which makes this case a spatial chronosequence of FBT in a time scale of a few decades in aapa mires. Furthermore, the FBT in this case connects to climate warming and the climate zonation of the focal mire types, as the expanding bog vegetation represents a southern element. Warming‐related changes can onset FBT in boreal mires (Kolari et al., 2022; Tahvanainen, 2011), providing one potential mechanism to explain the increase of carbon sequestration in northern mires, as projected for recent past and future (Gallego‐Sala et al., 2018).

In Europe, aapa mires prevail in northern and middle boreal zones, while raised bogs predominantly occur in southern and hemi‐boreal zones (Eurola & Vorren, 1980). The occurrence of aapa mires is largely explained by thermal factors, snow cover, and discharge patterns (Heikkinen et al., 2022; Parviainen & Luoto, 2007; Sallinen et al., 2023). According to Ruuhijärvi (1960), aapa mires predominantly occur north of the isocline of effective temperature sum of 1100°C, but this isocline has moved northwards since his classic work (Sallinen et al., 2023). In northern Finland, the effective temperature sum is projected nearly to double from the 1971–2000 average (Ruosteenoja et al., 2011), and thermal summer may lengthen by 30 days by the period 2040–2069 under the RCP4.5 scenario (Ruosteenoja et al., 2020). Thus, the climate conditions suitable for aapa mires, particularly string‐flark fen habitats, are soon lost in the southern range margin (Heikkinen et al., 2022), remarkably affecting the hydrology of aapa mires (Sallinen et al., 2023). Given that supplementary mineral input from the catchment and flooding by snowmelt water maintain the minerotrophic fen vegetation and string‐flark patterning of aapa mires, the projected decline of peak flow after snowmelt may induce changes in aapa mires.

One mechanism of FBT, as recently recognized in several Finnish aapa mires, is a lateral expansion of *Sphagnum*‐dominated bog margins over central fens (Granlund et al., 2022; Kolari et al., 2022). This phenomenon differs from the lateral expansion of mire vegetation over surrounding dry mineral soil, i.e., paludification (Bauer et al., 2003; Kuhry & Turunen, 2006), and it can rather be considered as a type of hydroseral development, initiating as infilling of flarks by floating Sphagna and eventually leading to terrestrialization as bog ecosystem (Granlund et al., 2022). This process in aapa mire complexes likely differs from the mid‐Holocene FBTs where transitional stages have often been associated with relatively dry poor‐fen vegetation with *Eriophorum vaginatum* (Hughes & Barber, 2003; Hughes & Dumayne‐Peaty, 2002; Tuittila et al., 2007; Väliranta et al., 2017). The ongoing expansion of *Sphagnum* bogs in aapa mire complexes provides an excellent opportunity to study vegetation succession along a spatial chronosequence, as we aim at revealing details of the ongoing and potential future succession trajectories.

In this study, we explore how plant community diversity and structure, and distribution of plant species and traits are altered with the FBT in aapa mires. We collected vegetation and water chemistry data from 23 undrained aapa mire complexes in the northern boreal zone of Finland in 2019–2020. Sampling was focused on sites where a priori comparisons of historical (1944–1970) and new (2017–2019) aerial photographs indicated recent lateral expansions of bog zones by an increase in *Sphagnum* cover at the transition zones between string‐flark fens and bog zones. In all study sites, vegetation plot data were collected from (1) the string‐flark fen, (2) the transition zone with an indication of *Sphagnum* increase, and (3) the bog zone, representing the FBT chronosequence phases. In the three spatial zones, plots were recorded as pairs located on two microhabitats: (1) a hummock string and (2) a flark area between strings (lawns in bogs). In a previous study, we used a multiproxy approach including a detailed analysis of dated peat stratigraphies to verify recent FBTs at five out of the 23 sites studied here (Granlund et al., 2022). Therefore, we are confident that our sampling represents a chronosequence of FBT, although we have not included peat analyses in this study. Instead, we aim to amend the picture of vegetation community and hydrological changes during FBT in aapa mires by including more study sites, thus, potentially covering more variation in FBT, some of which may result from various timing of the phenomenon.

Specifically, we ask (1) what is the succession trajectory of aapa mire complexes in the present climate, or are there multiple successional pathways, (2) can plant communities of modern FBT be recognized and what are their main characteristics (species, traits), (3) how water chemistry and water‐table depth (WTD) reflect fen‐bog transition, and (4) how fen‐bog transition alters the diversity patterns of mire complexes.

## 2 MATERIALS AND METHODS

### 2.1 Study area and site selection

The study sites (*n* = 23) locate in the north and middle boreal zones of Finland (Table 1). All study sites are characteristic aapa mire complexes, with minerotrophic central fen area and ombrotrophic bog vegetation at the mire margins (Ruuhijärvi, 1960). In the northernmost study site (Hämeenjänkä, Kemijärvi), the mean annual temperature (MAT) for the period 1961–2020 was 0.1°C and the average annual precipitation sum was 562 mm (Finnish Meteorological Institute, *open data*). During the same period, MAT increased by 0.4°C per decade and annual precipitation sum by 16 mm per decade. In the southernmost site (Kalattomansuo, Ilomantsi), MAT was 2.5°C and the average annual precipitation sum was 671 mm. From 1961 to 2020, MAT increased by 0.3°C per decade and annual precipitation sum by 7 mm per decade.

**TABLE 1:** Descriptions of study sites.

No.	Site name	Lat.	Long.	Altitude	Slope	Plots (*n*)	Disturbance	*Sphagnum*
1	Hämeenjänkä (East)	66°42′49″	27°59′9″	160	0.2	6	1	1
2	Hämeenjänkä (West)	66°43′3″	27°58′15″	158	0.4	6	1	1
3	Pekkasuo (East)	65°41′31″	25°29′50″	80	0.1	6	2	1
4	Pekkasuo (West)	65°41′29″	25°28′50″	80	0.1	6	3	0
5	Tammanlamminsuo	65°26′15″	28°7′57″	243	0.3	6	0	2
6	Saarisuo	65°25′55″	28°7′55″	243	0.2	6	0	2
7	Mäntysuo	65°25′49″	28°17′27″	226	0.3	6	1	1
8	Mäntylamminsuo	65°25′47″	28°15′52″	232	0.2	6	1	0
9	Kalettomansuo	65°25′9″	28°8′8″	235	0.5	6	1	0
10	Isonkivensuo	65°24′57″	28°17′48″	230	0.3	6	2	1
11	Olvassuo	65°6′41″	27°4′59″	119	<0.1	6	2	3
12	Jakosuo	64°56′55″	26°10′58″	75	<0.1	6	1	2
13	Susisuo	64°55′41″	26°10′41″	75	0.1	6	2	2
14	Maaselänsuo	64°43′21″	26°39′20″	105	0.1	7	3	1
15	Säippäsuo (West)	64°42′42″	26°41′56″	125	0.2	6	1	3
16	Hirsikankaansuo	64°42′33″	26°39′47″	110	0.7	6	3	2
17	Säippäsuo (South)	64°41′57″	26°43′42″	125	0.1	6	3	2
18	Leikonsuo	64°41′10″	26°39′30″	105	0.2	4	2	1
19	Haisunsuo	64°39′21″	26°1′15″	70	0.3	6	2	2
20	Pieni Sarvikangas	64°41′8″	25°54′49″	60	0.1	6	2	2
21	Ilajansuo	62°55′14″	31°12′41″	172	0.1	12	1	3
22	Viitasuo	62°49′16″	30°37′19″	145	0.1	12	0	3
23	Kalattomansuo	62°34′13″	30°53′1″	163	0.3	12	2	2

*Note*: Disturbances potentially affecting mire hydrology and a degree of *Sphagnum* increase were subjectively assessed from historical and new aerial photographs and scaled from 0 to 3. Disturbance: 0 = no effective disturbance, may have clear‐cutting in the catchment, 1 = minor disturbance, ditching likely not affecting, 2 = weak to moderate disturbances, ditching likely affecting runoff threshold but not incoming water, and 3 = moderate to strong disturbances, ditching likely reducing incoming water. *Sphagnum* increase: 0 = from no clear increase to only subtle indication, 1 = minor increase in *Sphagnum* cover, 2 = clear indication of an increase in *Sphagnum* cover, and 3 = indication of a large‐scale increase in *Sphagnum* cover.

**FIGURE 2:**
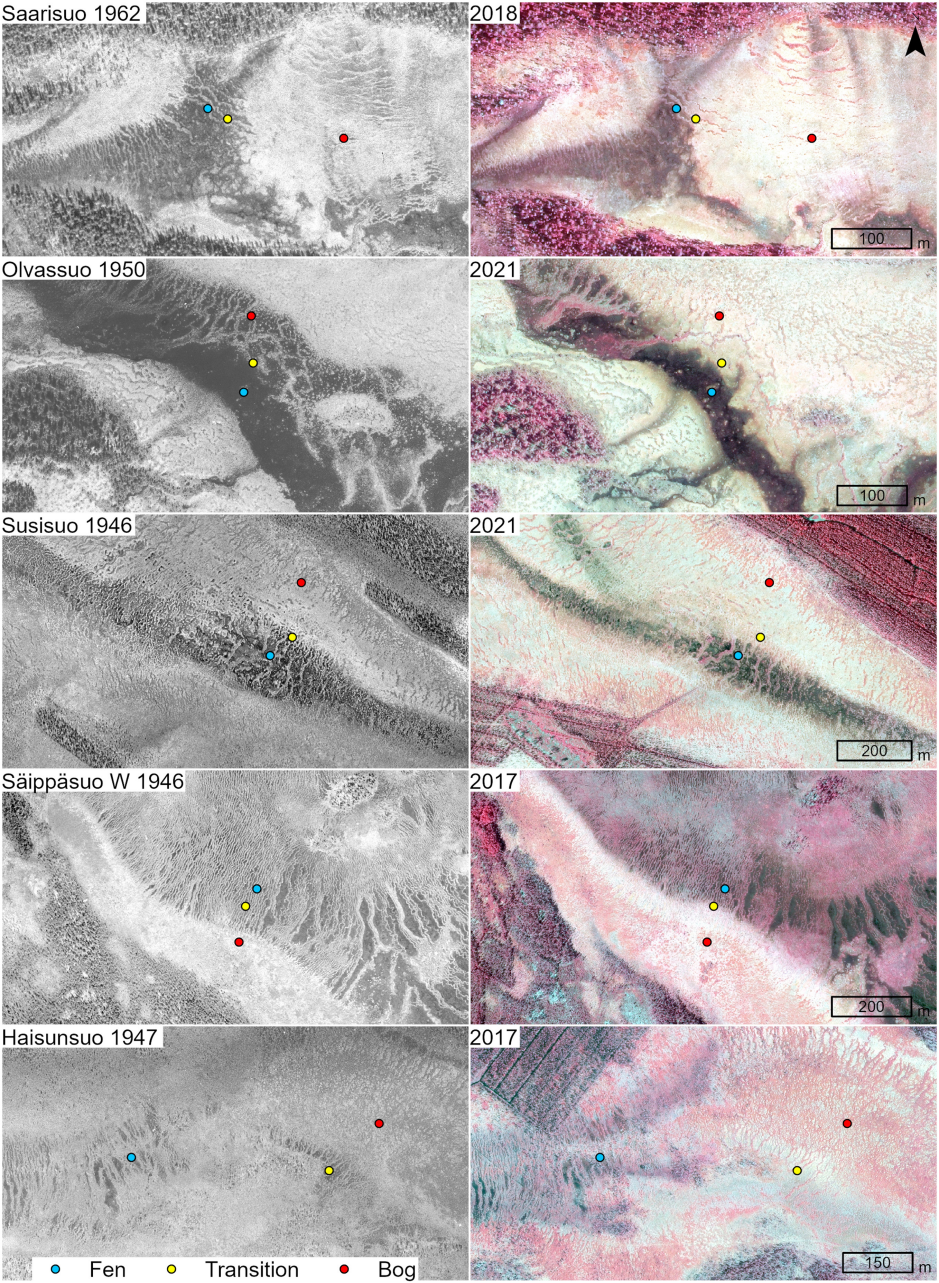
Historical (left column) and new (right column) aerial photographs for five out of 23 study sites, showing the varying degrees of *Sphagnum* expansion. In the historical photographs, wet fens are dark gray and black, and Sphagnum‐dominated carpets and lawns appear in light gray and whitish tones. New images are color‐infrared photos, where *Sphagnum* mosses appear in pale tones of green and yellow, and green vascular plant vegetation appears in red. Field data were collected from string‐flark pairs in fen, transition, and bog zones. Pairs of historical and new aerial photographs for each site are provided in Appendix S1. Source of aerial photographs: National Land Survey of Finland (open data).

The sites were selected by comparing historical aerial photographs from 1944–1970 to new photographs from 2017 to 2019 (Figure 2). Field sampling was focused on the sites, where aerial photograph comparisons indicated an increase of *Sphagnum* moss cover in central fens. Historical aerial photographs have proved to be an appropriate tool for studying the expansion of *Sphagnum* bogs over wet fens (Granlund et al., 2022; Kolari et al., 2022; Tahvanainen, 2011). In black‐and‐white photographs, *Sphagnum* mosses appear in whitish tones, in contrast with dark tones of flark fens with sparse or submerged vegetation. In this study, the degree of *Sphagnum* increase and disturbances in the mire catchments, potentially altering hydrology, were subjectively assessed from historical and new aerial photographs, and scaled from 0 to 3 (Table 1). The degree of *Sphagnum* increase varied between sites from an extensive lateral expansion of bog zone to minor changes in *Sphagnum* cover, and in a few cases, aerial photograph comparisons indicated only subtle changes that were difficult to disentangle from seasonal changes. All study sites are undrained, but disturbances in the catchment varied from no effective to strong disturbances, including mainly forestry ditching and clear‐cutting. Pairs of historical and new color‐infrared aerial photographs for each site are provided in Appendix S1. Hereinafter, we refer to the area with an indication of *Sphagnum* increase as a “transition” zone.

### 2.2 Field sampling

The study sites were visited in the summer of 2019 and 2020. In each study site, the cover (%) of each plant species was estimated in six locations (*n* = 155), with some exceptions (Table 1). Most often, data were collected from flark‐string pairs as triplets of samples from a minerotrophic string‐flark fen, a transition zone, and an ombrotrophic bog of the same mire complex. Technically, plot pairs in bogs located in bog lawns and hummocks, but with flarks and strings, we refer to micro‐topographical patterns in all mire zones, if not specified otherwise.

Precise locations of pairs within the mire zones were randomly selected in the field. In each location, coverages of plant species were estimated from a phytosociological relevé and from a nested subplot of 0.25 m^2^. The purpose of phytosociological relevés was to catch whole species assemblages, thus, the size of the relevé depended on the area of the micro‐topographical pattern. The relevé size varied between 2 and 25 m^2^ but was most often 16 m^2^. In relevés, the abundance scale following the Braun‐Blanquet approach was used. In subplots, coverages were estimated on a continuous scale from one to 100%.

Water‐table depth (WTD) was measured in each plot as a distance from the water level to the top of the bryophyte layer. Water pH was measured in the field with a portable pH/conductivity tester (Consort). A water sample of 50 mL was obtained in each plot for chemical analyses using a 50‐cm long perforated plastic pipe well (*n* = 145), although some bog hummocks had too deep water table for obtaining a sufficient water sample.

### 2.3 Vegetation data analyses

#### 2.3.1 Species distribution patterns

We used indicator species analysis to determine habitat‐association patterns of species between the six micro‐topographical patterns of the fen‐bog chronosequence: fen flarks, fen strings, transition flarks, transition strings, bog lawns, and bog hummocks. Indicator species analysis was performed with the 0.25‐m^2^ vegetation plot data using function multipatt in the package *indicspecies* (De Cáceres & Legendre, 2009) in R version 4.1.1 (R Core Team, 2021) and with 4999 permutations within blocks (study sites). This function allows studying the associations of species to combinations of habitat groups, in addition to specific habitat types. We used the Indicator Value (IndVal) index of Dufrêne and Legendre (1997), a method that considers both relative abundance and relative frequency of a species in habitat groups and then looks for the group with the highest association value.

#### 2.3.2 Plant community composition turnover

Differences in plant community structure between mire zones were further examined through nonmetric multidimensional scaling (NMDS) of the relevé data, using function metaMDS in the package *vegan* 2.5‐7 (Oksanen et al., 2020). NMDS was run separately for bryophyte and vascular plant communities of flarks and strings, using nontransformed data, Bray–Curtis dissimilarity values between plots, a maximum of 200 iterations, and a 2‐dimensional solution.

A permutation‐based nonparametric multivariate analysis of variance (PERMANOVA) was used to test the difference in bryophyte and vascular plant communities between the mire zones using the relevé data. PERMANOVA analyses were run in PC‐ORD 7.08 (McCune & Mefford, 2018) and separately for flarks and strings. To get balanced data with six measurements from each study site, 132 out of total 155 plots were included in PERMANOVA (66 flark and 66 string plots). In the analyses, the site was treated as a block factor and zone as the grouping factor, and Bray–Curtis dissimilarity values were used. PERMANOVA was run for nine different datasets (all species, bryophytes, vascular plants, aerenchymatous, and nonaerenchymatous species of flarks and strings). The dataset with nonaerenchymatous species of flarks had several empty rows (zero species in a plot); therefore, PERMANOVA was not performed in that case.

Additionally, we calculated mean Bray–Curtis dissimilarity values within zones and between zones for pairwise comparisons (fen vs. transition, fen vs. bog, and bog vs. transition) using the relevé data. This was done in R version 4.1.1 (R Core Team, 2021), using functions vegdist and meandist in the package *vegan* 2.5‐7 (Oksanen et al., 2020). By comparing mean dissimilarity values, we aimed at (1) examining the level of variation within mire zones between sites and (2) discovering whether transitional communities are more alike with fen or bog communities. Within mire zones, a low mean dissimilarity value indicates low variation and a high‐value large variation among study sites.

#### 2.3.3 Plant functional groups and diversity

We explored differences in species richness, Shannon diversity index, and the total cover and number of species of bryophytes and vascular plants. As species with root aerenchyma are common in wet habitats and capable of transporting methane from peat to the atmosphere (Korrensalo et al., 2022), vascular plants were further divided into shallow‐ and deep‐rooted, and aerenchymatous and nonaerenchymatous species, based on the classic work of Metsävainio (1931) with the full catalog of root traits of wetland plants. Aerenchymatous species include mainly sedges, such as *Carex* spp. and *Eriophorum* spp. Other examples of aerenchymatous species of different clades are *Equisetum fluviatile*, *Menyanthes trifoliata*, *Rubus chamaemorus*, and *Scheuchzeria palustris*. Nonaerenchymatous species consisted mainly of shrubs, including *Andromeda polifolia*, *Betula nana*, *Empetrum nigrum*, *Chamaedaphne calyculata*, *Rhododendron tomentosum*, and *Vaccinium* spp.; and forbs, such as *Drosera* spp. and *Utricularia* spp.

Normality of the data was assessed from histograms, and a logarithmic transformation was applied to all cover values to approach normality. Additionally, normality of residuals was assessed from histograms and using the Shapiro–Wilk normality test, and the assumption of normality was accepted in all cases. Differences between zones (fen, transition, and bog) and micro‐topographic patterns (flarks and strings), and their interactions were tested using a general linear model with zone and pattern as fixed factors and mire as a random factor. Tukey HSD post hoc tests were separately performed for strings and flarks to study pairwise differences between zones. General linear models and post hoc tests were run in SPSS Version 27 and using data from subplots of 0.25 m^2^.

Beta diversity was defined for each string‐flark pair (subplots, *n* = 66) as gamma diversity/alpha diversity, where gamma is the total species number and alpha mean species number in the two subplots. Differences in beta diversity between mire zones were tested using Wilcoxon signed rank test and function compare_means in the package *ggpubr* (Kassambara, 2020) in R version 4.1.1. (R Core Team, 2021).

Plant species nomenclature follows the Finnish Biodiversity Info Facility (www.laji.fi, 7.11.2022).

### 2.4 Water chemistry and water table

Water samples were analyzed for dissolved organic carbon (DOC) and 22 mineral elements, but several mineral elements were under or close to detection limits and were omitted from the statistical analyses. Before DOC and elemental analyses, samples were filtered through 0.45 μm sterilized membrane (Pall Corporation) or syringe filters (VWR International). Subsamples for DOC analysis (10–15 mL) were analyzed with a multi N/C® 2100 TOC analyzer (Analytik Jena AG) within 2 months after sampling. Subsamples for elemental analysis (10–15 mL) were analyzed by Inductively Coupled Plasma—Mass Spectrometry (ICP‐MS) using a NeXION 350D ICP‐MS instrument (PerkinElmer Inc.). Multi‐element standard solution (TraceCERT® Periodic table mix 1 for ICP, Sigma‐Aldrich) was used for the calibration of ICP‐MS.

We compared pH, DOC, and concentrations of six mineral elements (Al, Ca, Fe, Mg, Mn, and Si) between the mire zones (fen, transition, and bog). Average per zone was used in the comparisons (*n* = 68) because two to four water samples were collected per zone in each site. Normality of the data was assessed from Q–Q plots and using the Shapiro–Wilk normality test (shapiro.test, package *stats*) in R version 4.1.1 (R Core Team, 2021). As the assumptions of normal distribution did not hold in most comparisons, we tested the differences in pH, DOC, and mineral element concentrations using Wilcoxon signed rank test with Holm‐adjusted *p*‐values, using function compare_means in the package *ggpubr* (Kassambara, 2020). The difference in WTD was similarly tested between zones, but flarks and string hummocks were treated separately.

## 3 RESULTS

### 3.1 Species distribution patterns

**FIGURE 3:**
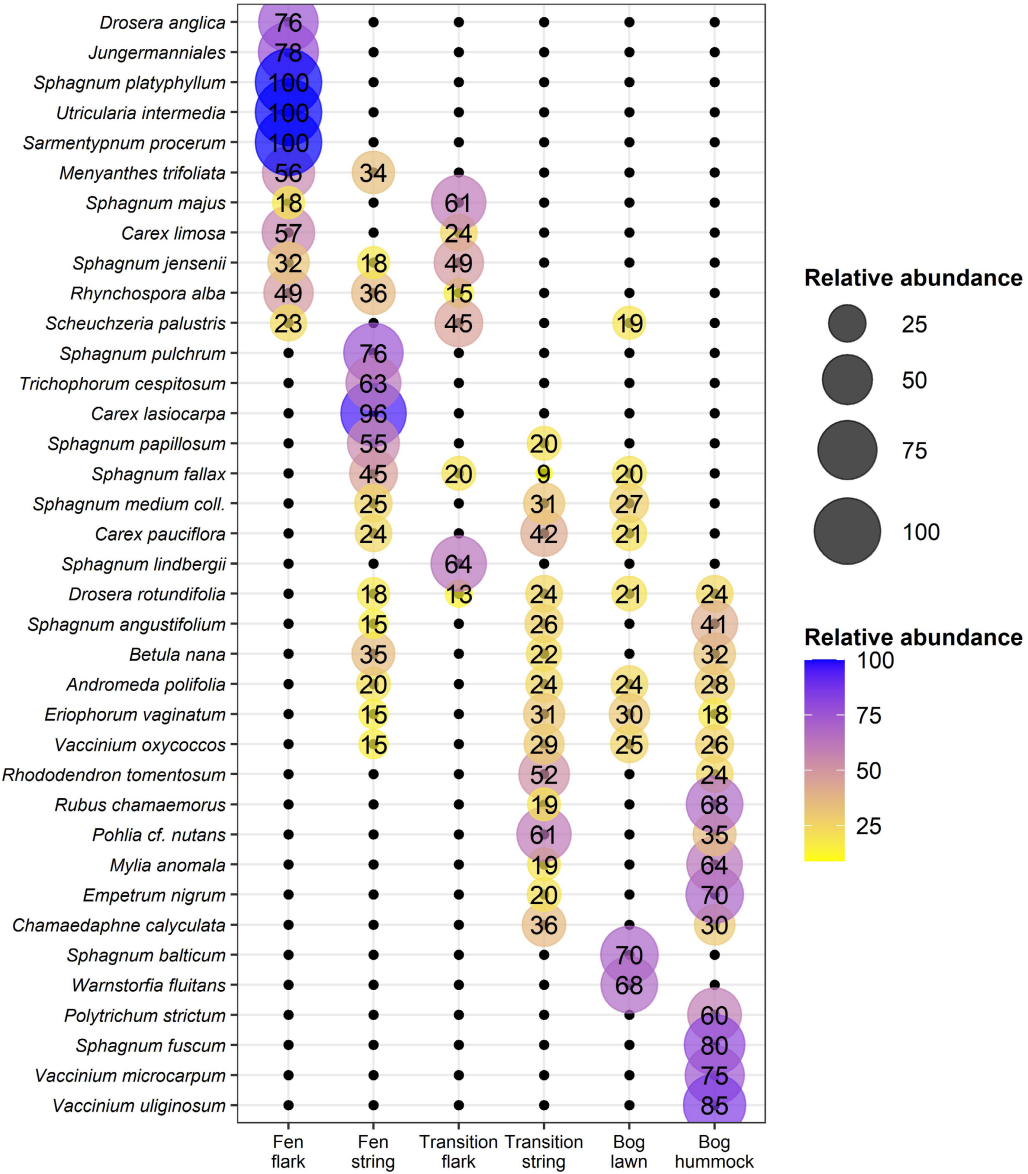
Significant (*p* < .05) indicator species and their relative abundances in six micro‐topographical patterns. Relative abundances are shown only for the patterns where the species showed significant association, although species may have occurred in other micro‐topographical patterns as well.

Among the 66 species, 37 (56%) showed significant habitat associations in the indicator species analysis (Figure 3). Of these, 15 species showed significant (*p* < .05) associations to single microhabitats, ten species were associated with combinations of two microhabitats, seven species were associated with three microhabitats, four species with four microhabitats, and one species with five microhabitats.

Several strong indicators were found for the fen flarks (*Drosera anglica*, *Sphagnum platyphyllum*, *Utricularia intermedia*, and *Sarmentypnum procerum*), fen strings (*Sphagnum pulchrum*, *Trichophorum cespitosum*, and *Carex lasiocarpa*), bog lawns (*Sphagnum balticum*, *Warnstorfia fluitans*), and bog hummocks (*Polytrichum strictum*, *Sphagnum fuscum*, *Vaccinium microcarpum*, and *Vaccinium uliginosum*). *Sphagnum lindbergii* was the only species to show a significant association only with the transitional flarks, while the transitional strings had no distinct indicators. Six species were indicative of all hummock surfaces, irrespective of mire zones, while only one flark species (*Scheuchzeria palustris*) had a joint indication of all wet surfaces.

### 3.2 Plant community compositional turnover

**FIGURE 4:**
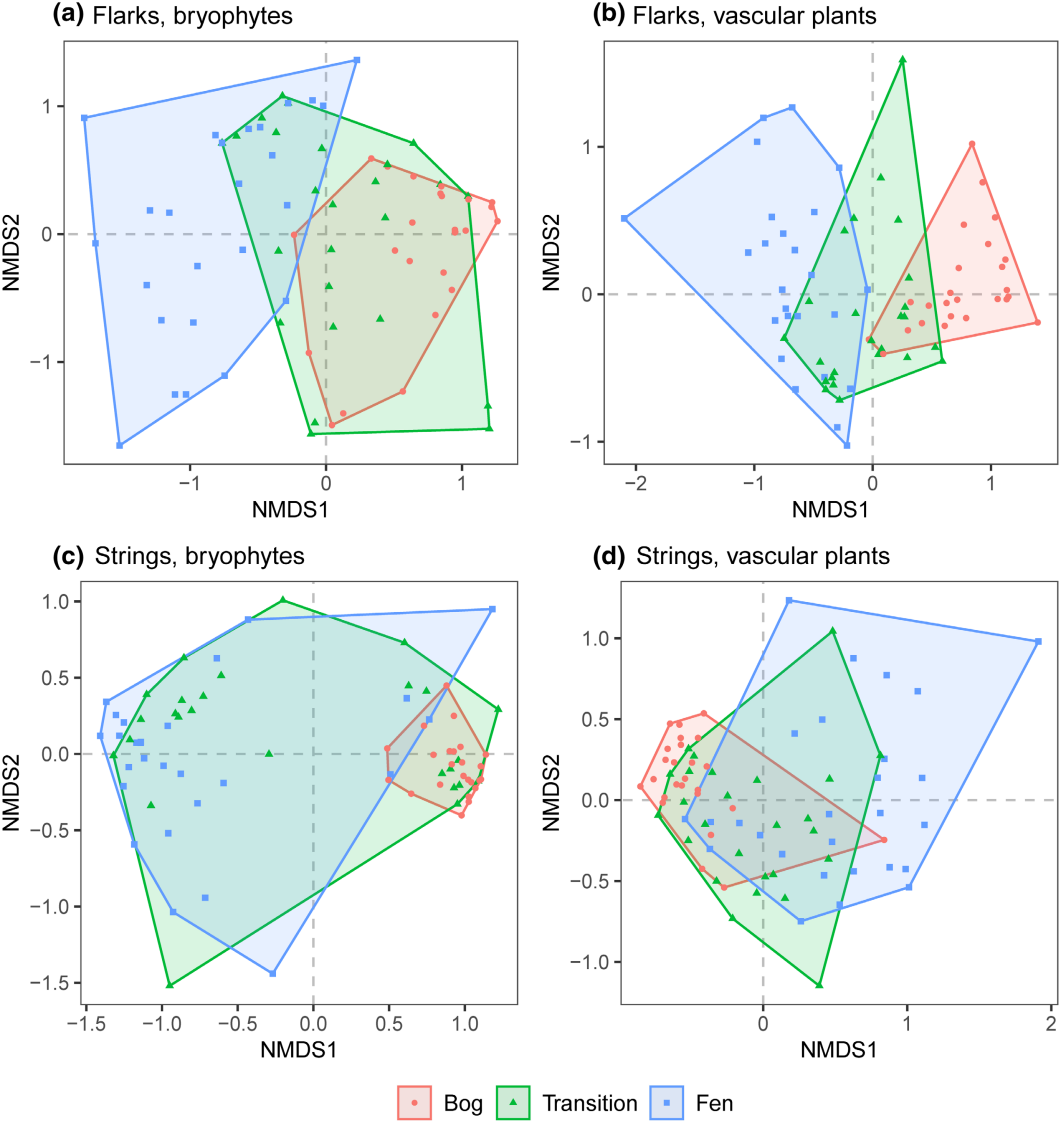
The nonmetric multidimensional scaling (NMDS) ordinations of vegetation plots: (a) bryophyte communities of flarks (stress: 0.179), (b) vascular plant communities of flarks (stress: 0.163), (c) bryophyte communities of strings (stress: 0.114), and (d) vascular plant communities of strings (stress: 0.188). Ordinations show a clear separation of fens and bog plant communities, while transitional communities have a varying degree of overlap with both fen and bog communities.

The NMDS ordinations of species abundance data generally illustrated the separation of fen and bog communities, with little overlap, and the transition zone samples formed intermediate groups with varying overlap with the fen and bog communities (Figure 4). The ordinations of the bryophyte communities and the vascular plant communities of flarks (Figure 4a, b), both showed gradients from wet, poor‐fen communities to drier ombrotrophic bog vegetation. Transitional bryophyte communities had more extensive overlap with bogs than with fen communities, while transitional vascular plant communities had approximately the same amount of overlap with fen flark and bog lawn communities.

In the case of the string hummocks, the ordination of bryophytes showed a clear separation of the fen strings and the *Sphagnum fuscum*‐dominated bog hummocks, with some exceptional sampling points (Figure 4c). The fen string plots were distributed along the NMDS Axis 2, predominantly on the left‐hand side of the ordination space, while bog hummocks had little variation, forming a compact group on the right. Transitional strings were similar to either fen strings or bog hummocks. The ranges of vascular plant communities of fens, transition zones, and bogs were all overlapping the ordination space (Figure 4d). Again, fen string plots were widely distributed along both NMDS Axis 1 and 2, while the bog hummock plots were predominantly located in the lower left corner of the ordination space.

The pairwise PERMANOVA confirmed that the bryophyte and vascular plant communities of both flarks and strings differed significantly between all mire zones (Table 2). Significant differences were also found when datasets with full species assemblages were used. PERMANOVA was also run separately for the abundance data of aerenchymatous and nonaerenchymatous species of flarks and strings. Pairwise comparisons showed significant differences between all mire zones in each case, except for nonaerenchymatous species of flarks that were not determined due to several empty plots in fens and transition zones.

**TABLE 2:** Mean Bray–Curtis dissimilarities of vegetation plots within and between zones.

	Fen	Transition	Bog	*p*	Pairwise comparisons					
					Fen vs. bog	*p*	Fen vs. transition	*p*	Bog vs. transition	*p*
Flarks										
All species	0.709	0.656	0.532	***	0.898	***	0.742	***	0.736	***
Bryophytes	0.796	0.700	0.555	***	0.933	***	0.805	***	0.760	***
Vascular plants	0.614	0.535	0.476	***	0.854	***	0.639	***	0.685	***
Aerenchymatous spp.	0.575	0.485	0.513	***	0.834	***	0.592	***	0.674	***
Nonaerenchymatous spp.	nd	nd	nd	nd	nd	nd	nd	nd	nd	nd
Strings										
All species	0.652	0.606	0.387	***	0.793	***	0.680	***	0.586	***
Bryophytes	0.658	0.715	0.227	***	0.879	***	0.721	**	0.663	***
Vascular plants	0.644	0.557	0.398	***	0.721	***	0.644	***	0.547	***
Aerenchymatous spp.	0.718	0.547	0.372	***	0.800	***	0.715	***	0.514	***
Nonaerenchymatous spp.	0.503	0.518	0.489	***	0.635	***	0.539	*	0.539	***

*Note*: *p*‐Values for pairwise comparisons are derived from PERMANOVA with the site as a blocking and zone as a grouping variable (***p < .001, **p < .01, and *p < .05). Bray–Curtis dissimilarities were calculated using different datasets consisting of all species, bryophytes, vascular plants, aerenchymatous vascular plants, and nonaerenchymatous vascular plants. This was done separately for flarks and strings. PERMANOVA was not run in the case of nonaerenchymatous species of flarks due to several empty plots in fens and transition zones (nd = not determined).

Within the mire zones, mean Bray–Curtis dissimilarity values were the highest in the fens and the lowest in the bogs (Table 2). This applied to full species assemblages, and in separate assessments of bryophyte and vascular plant communities of both flarks and strings, as well as for aerenchymatous and nonaerenchymatous species. These values indicated greater variation within the fens, as compared to the transitional and bog communities. However, in the case of aerenchymatous species of flarks, the mean Bray–Curtis dissimilarity value was the lowest (0.485) within transition zones.

In pairwise comparisons, the mean Bray–Curtis dissimilarity values were the highest between the fens and the bogs in each case, as indicated also by the NMDS ordinations. When the transitional communities were compared with the fen and bog communities, mean dissimilarity values were most often lower in the comparisons with the bogs than with fens. However, in the case of vascular plant communities and aerenchymatous species of flarks, mean dissimilarity values were lower in the comparisons with fens. Nonaerenchymatous species of strings had the same mean dissimilarity value in both comparisons.

### 3.3 Plant functional groups and diversity

In total, 78 species were recorded in the study sites (36 bryophytes, 38 vascular plants, and four lichens). When both flark and string plots were included in ANOVA, significant differences between mire zones were found in overall species richness (*p* = .035), and the number of all vascular plant species (*p* = .002), nonaerenchymatous (*p* < .001), and aerenchymatous species (*p* = .004; Table 3). Significant differences were also found in the coverages of bryophytes (*p* = .001), nonaerenchymatous species (*p* < .001), and shallow‐ and deep‐rooted aerenchymatous species (*p* < .001 and *p* = .022, respectively). In the cases of species richness, bryophyte cover, and the number of bryophytes, all vascular plants, and aerenchymatous species, the response depended on the pattern (significant interaction with *p* < .01). On average, species richness and the covers of bryophytes, all vascular plants, and nonaerenchymatous species were highest in the bogs, while the cover and number of aerenchymatous species were highest in the fens.

**TABLE 3:** Results from ANOVA with mire zone and micro‐topographical pattern as fixed factors and site as a random factor (results for sites not shown).

	Means for zones			Zone		Pattern		Zone × pattern	
	Fen	Transition	Bog	*F*	*p*	*F*	*p*	*F*	*p*
Species richness (S)	8.1	9.0	9.2	3.442	.035	95.234	.000	8.351	.000
Shannon index (H′)	1.273	1.280	1.148	1.310	.273	8.970	.003	0.689	.504
Number of bryophyte spp.	2.9	3.2	2.9	1.284	.281	8.561	.004	5.330	.006
Bryophyte cover	36.8	52.9	66.7	7.047	.001	17.909	.000	8.711	.000
Number of vascular plant spp.	5.2	5.8	6.3	6.356	.002	114.217	.000	5.473	.005
Vascular plant cover	19.9	21.3	24.8	2.206	.114	68.737	.000	0.013	.987
Number of nonaerenchymatous spp.	2.2	2.8	4.0	25.875	.000	182.211	.000	1.509	.225
Nonaerenchymatous cover	6.8	9.1	14.1	21.574	.000	133.604	.000	2.922	.057
Number of aerenchymatous spp.	3.0	2.9	2.3	5.771	.004	0.604	.438	8.474	.000
Aerenchymatous cover	13.1	12.2	10.7	1.414	.247	2.883	.092	0.333	.717
Shallow‐rooted cover	3.4	5.6	1.6	13.566	.000	25.245	.000	2.068	.131
Deep‐rooted cover	9.7	6.6	9.0	3.953	.022	25.067	.000	0.074	.929

*Note*: Zone refers to fen, transition zone, and bog. Pattern means flarks and strings. Significant *p*‐values (<.05) are highlighted.

**FIGURE 5:**
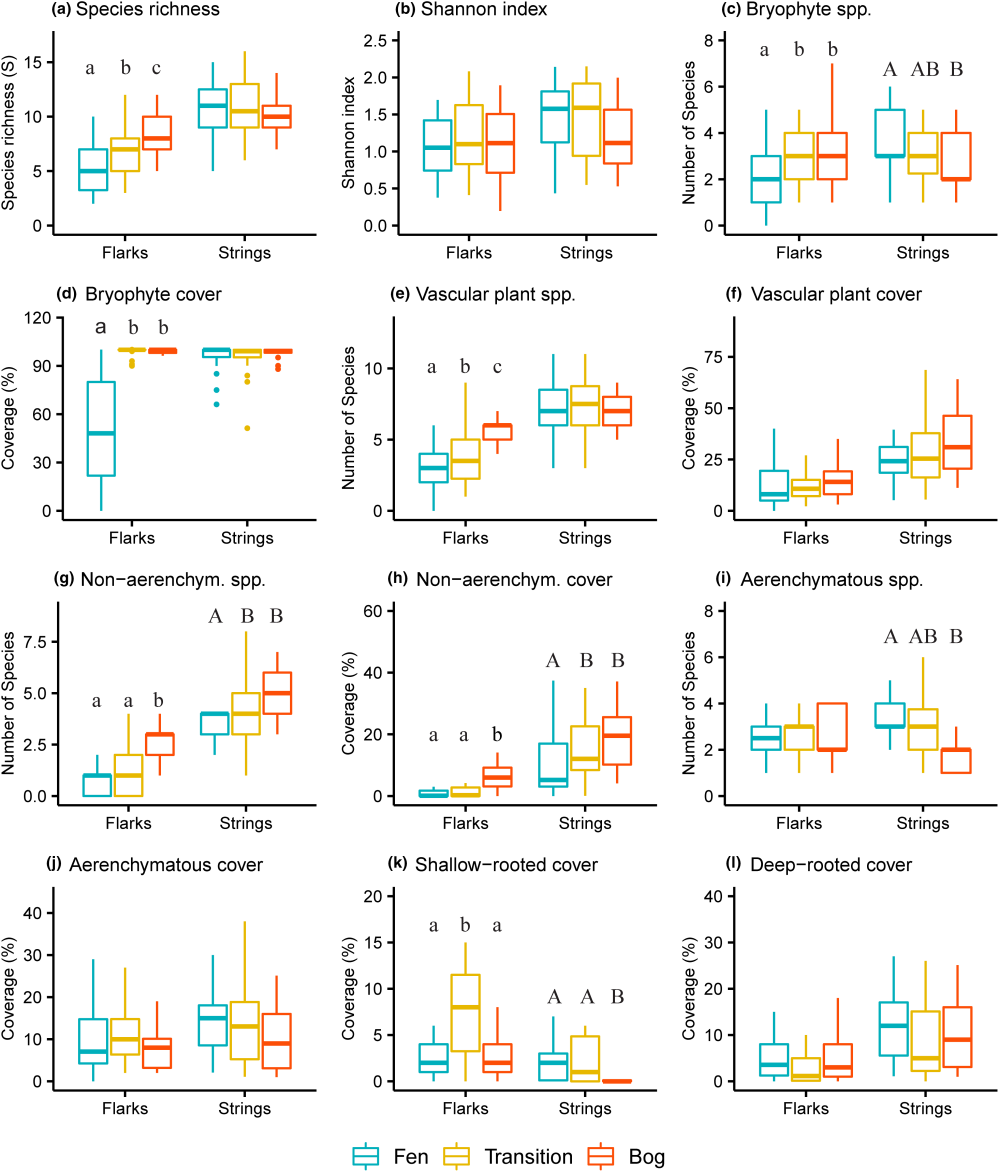
Number of species and total cover of plant groups of flarks and strings in fens, transition zones, and bogs. *p*‐values for pairwise comparisons of mire zones were derived from Tukey HSD post hoc tests. Outliers were removed from the figures, except in the case of bryophyte cover.

On flarks, pairwise comparisons showed that overall species richness and a number of vascular plant species were lowest in fens and increased towards bogs (Figure 5). The number of bryophyte species was also significantly higher in bogs and transition zones compared with fen flarks, although it was generally very low (2.7 species on average). The cover of bryophytes was significantly higher in the transition zones and bogs than in fen flarks, while no significant difference was found between transition zones and bogs. The number and cover of nonaerenchymatous species were significantly higher in bogs than in fens and transition zones. The cover of shallow‐rooted aerenchymatous species was significantly higher in the transition zone compared with both fens and bogs, while no significant differences were found in the cover of deep‐rooted species. No significant differences were found either in the Shannon diversity index, the number and cover of aerenchymatous species, and the cover of all vascular plants.

On strings, pairwise comparisons showed significant differences in the number of bryophytes, aerenchymatous, and nonaerenchymatous vascular plant species, and the coverages of nonaerenchymatous and shallow‐rooted aerenchymatous species. The number of bryophyte species was significantly higher in fen strings (mean 3.7) compared with bog hummocks (mean 2.8). The number and cover of nonaerenchymous species were significantly higher in transition zones and bogs than in fens, while transition zone did not differ significantly from bogs. The number of aerenchymatous species was significantly lower in bogs than in transition zones and fens, but the cover did not differ significantly between zones. The cover of shallow‐rooted species was significantly higher in fen strings than in bog hummocks.

**FIGURE 6:**
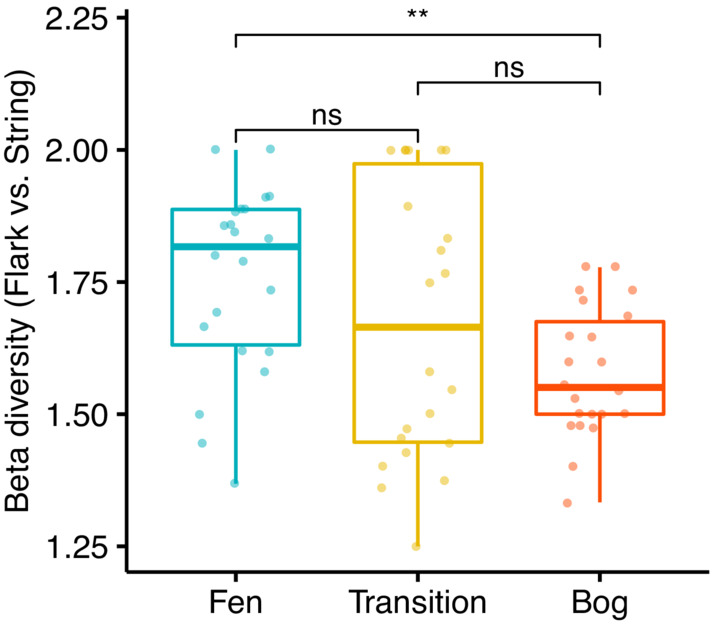
Beta diversity in fens, transition zones, and bogs. Beta diversity was defined for each string‐flark pair as gamma diversity/alpha diversity, where gamma is total species number in the plot pair, and alpha is mean species number per the plot pair. *p*‐values for pairwise comparisons were derived from Wilcoxon signed rank test.

Beta diversity, as defined for each string‐flark pair, was significantly higher in fens than in bogs (*p* < .0024; Figure 6). Along the fen‐bog gradient, beta diversity decreased towards bogs, but no significant differences were found either between transition zones and fens (*p* = .0554) or between transition zones and bogs.

### 3.4 Water chemistry and water table

**FIGURE 7:**
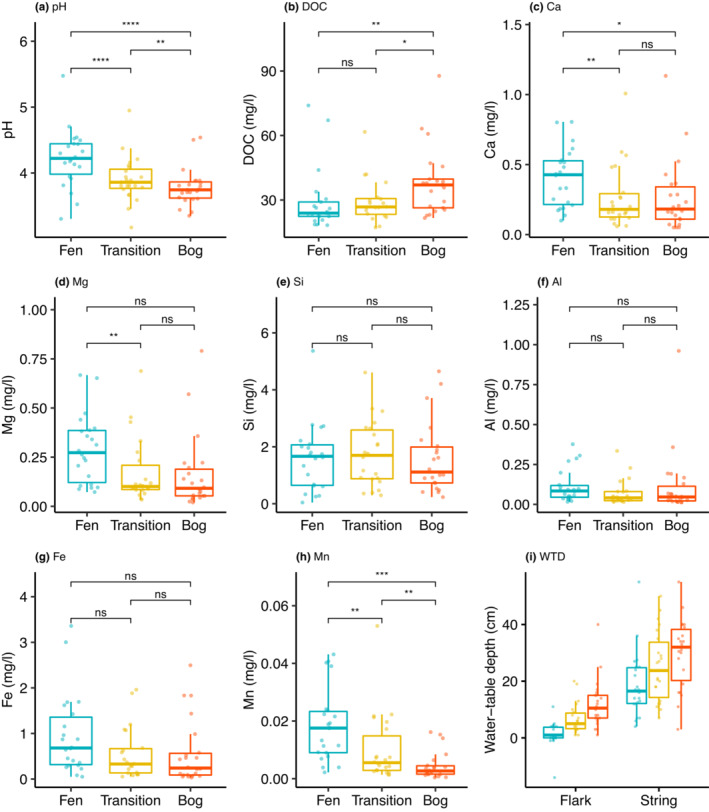
a) Water pH, b) dissolved organic carbon (DOC), c*p*h) concentrations of mineral elements, and i) water‐table depth (WTD) in fen, transition, and bog zones. Averages of flark‐string pairs are presented for water chemical variables. WTD is presented for both flarks and strings. Significant *p*‐values for pairwise comparisons were derived from Wilcoxon Signed Rank test (ns: *p* > .05, *p ≤ .05, **p ≤ .01, ***p ≤ .001, and ****p ≤ .0001.

Generally, pH and all mineral element concentrations were low in all study sites. The string‐flark fen zones had the highest average pH (4.22, SD 0.45), Ca concentration (0.40 mg/L, SD 0.21), and Mg concentration (0.29 mg/L, SD 0.17). Between the mire zones, significant differences were found in pH, DOC, and concentrations of Ca, Mg, and Mn (Figure 7). In the transition zones, pH was significantly lower compared with fens (*p* < .001), and significantly higher than in the bogs (*p* < .01). In DOC, no significant difference was found between the fen and transition zones, while DOC was significantly higher in the bog than in the fen (*p* = .021) and the transition zones (*p* = .028). Concentrations of Ca, Mg, and Mn were significantly lower in the transition zones than in the fen zones (*p*‐values: .011, .015, and <.01, respectively). Ca and Mg concentrations did not differ significantly between the transition zones and the bog zones, while Mn concentrations were significantly higher (*p* < .01) in the transition zones. On average, concentrations of Al and Fe were highest in the fen zones, but no significant differences were found.

The average WTD was 1 cm (SD 4.3) in the fen flarks, 7 cm (SD 5.2) in the transition flarks, and 12 cm (SD 8.6) in the bog lawns (Figure 7i). On hummock surfaces, the average WTD was 19 cm (SD 11.4) in the fen strings, 25 cm (SD 12.4) in the transition strings, and 30 cm (SD 12.4) in the bog hummocks. Significant differences were found between all three wet surfaces (*p* < .001) and all hummock surfaces (*p* < .05).

## 4 DISCUSSION

### 4.1 Characteristics of transitional plant communities

Our study of spatial chronosequences in 23 boreal aapa mire complexes showed a fairly uniform pattern of fen‐bog transition (FBT) through a quaking moss carpet phase with an increase in *Sphagnum* section *Cuspidata*. The most abundant species in transitional flarks, in descending order, were *Sphagnum majus*, *Scheuchzeria palustris*, *Sphagnum balticum*, *S*. *papillosum*, *S*. *jensenii*, *S*. *lindbergii*, and *Eriophorum vaginatum*. *S*. *lindbergii* was indicative only of transitional flarks, but there were no species that only occurred in transitional communities. Indeed, Gałka et al. (2017) found *S*. *lindbergii* present in the transition phase in paleoecological reconstructions from a subarctic mire and suggested that *S*. *lindbergii* functions mainly as a transitional species during FBT due to its low competitivity. Although *S*. *lindbergii* does occur in ombrotrophic bogs as well, its pH range is centered around pH 4.4, higher than other bog Sphagna, and it rarely occurs above pH 5 (Johnson et al., 2015; Wojtuń et al., 2013). *S*. *lindbergii* is known for the rapid colonization of disturbed sites like thermokarst (Markkula & Kuhry, 2020) and peat‐cutting pits (Soro et al., 1999). The species assemblage of transitional flarks included many species that were also frequent in fen flarks (*Carex limosa*, *Rhynchospora alba*, *Sphagnum jensenii*, *S*. *majus*, and *Scheuchzeria palustris*) and fewer species that were shared with bog lawns (Figure 3). In transitional strings, the species assemblage included persisting fen string species (*S*. *papillosum* as the dominant) and many generalist hummock species (e.g., *Andromeda polifolia*, *Betula nana*, and *Vaccinium oxycoccos*). Most remarkably, many fen species had significantly decreased or were absent from transitional vegetation, including aquatic flark species (*Sphagnum platyphyllum*, *Utricularia intermedia*, and *Sarmentypnum procerum*) and sedges in strings (*Carex lasiocarpa*, *Trichophorum cespitosum*). Thus, the transitional character in vegetation resulted from an altered selection between species, instead of the dispersal of transitional habitat specialists.

Contrary to our expectation, deep‐rooted aerenchymatous sedges were rare in transitional vegetation and we did not find evidence of their persistence as “fen relicts” under the expansion of *Sphagnum* mosses. Instead, *Sphagnum* expansion had favored some shallow‐rooted aerenchymatous vascular plants. Most importantly, *Scheuchzeria palustris* was particularly abundant in transitional flarks, probably facilitated by the formation of quaking *Sphagnum* carpets (Laberge et al., 2015). *Scheuchzeria* has shallower roots compared with other frequent fen flark species, e.g., *Carex limosa* and *Menyanthes trifoliata* (Metsävainio, 1931), and as a typical *k*‐strategist, it has large but few seeds. Laberge et al. (2015) found that seed germination of *S*. *palustris* was higher on *Cuspidata* carpets (*S*. *cuspidatum*) compared with seedbeds of *Cladopodiella fluitans*, a common liverwort of fen flarks in aapa mires. As they justified, germination may be hindered on compact *C*. *fluitans* seedbeds due to the fact that they are difficult for large seeds to penetrate, while *S*. *Cuspidata* carpets are more porous and still have high water level, facilitating germination and better root development (Landry et al., 2012). It should be noted that *Scheuchzeria* rarely reestablishes spontaneously in restoration sites, despite the rapid increase in *Sphagnum*, because it is usually absent in the landscape when restoration measures initially take place (Haapalehto et al., 2011). In pristine aapa mires, *Scheuchzeria* is frequently present in fen flarks, allowing its rapid proliferation with expanding *Sphagnum* carpets. In our dataset, *Scheuchzeria* was indicative of all wet surfaces, and it differentiated transitional flarks mainly by higher abundance.

The use of spatial chronosequence allowed comparisons of plant community structure with full species assemblages, while in most paleoecological research, information is limited to fewer identifiable species or to the level of plant structural or taxonomical groups. As expected, transitional communities had features of both fens and bogs, in line with a previous chronosequence study conducted in a coastal land‐uplift region (Tuittila et al., 2013). However, bryophyte and vascular plant communities showed different patterns of succession stage and microtopography, which could be expected, as vascular plants reach the nutrients of deeper peat layers, depending on their root systems, while bryophytes are more dependent on water level and quality. Transitional flarks closely resembled bog lawns in bryophyte community structure, while vascular plant communities often still resembled fen communities (Figure 4). On transitional strings, bryophyte communities tended to have either aapa string or bog hummock vegetation, instead of having unique transitional character, and vascular plant communities were most often similar to bog hummocks.

In a level of habitat and vegetation types, a majority of transitional flark communities can be considered as noncalcareous quaking mires (Chytrý et al., 2020) that correspond to the alliance *Scheuchzerion palustris* (Peterka et al., 2017). Although this alliance is under the *Scheuchzerio palustris‐Caricetea fuscae* class of phytosociological classification of fen vegetation, corresponding vegetation is commonly found also in ombrotrophic bog hollows. The vegetation typical of transitional strings with *Sphagnum medium* coll., *S*. *papillosum*, *S*. *fallax*, *Eriophorum vaginatum*, and several shrub species (e.g., *Andromeda polifolia*, *Betula nana*, and *Vaccinium oxycoccos*) likely belongs to the alliance *Sphagnion medii* of classification of European bog vegetation (Jiroušek et al., 2022).

### 4.2 Past and future bog succession trajectories

Our findings suggest that in the present climate, bog succession through a wet phase with *Spahgnum* sect. *Cuspidata* is a likely future trajectory in boreal aapa mire complexes with weak minerotrophy. Fen flarks were characterized by liverworts (Jungermanniales, mainly *Cladopodiella fluitans* and *Gymnocolea inflata*), and indicators of intermediate fens (mesotrophic, sensu Eurola et al., 2015), including *Sarmentypnum procerum*, *Sphagnum platyphyllum*, *Menyanthes trifoliata*, and *Carex limosa*. However, species that had proliferated in transitional flarks (*S*. *majus*, *S*. *jensenii*, and *Scheuchzeria palustris*) were often present already in the flark fens and they continued to occur also in the bog lawns, where *S*. *balticum* became the dominant moss species. This trajectory reappeared in most of our study sites. Elsewhere in a rich fen, we found an increase in hummock *Sphagnum* instead of wet Sphagna (Kolari et al., 2021) and resemblance to that case was found in one of our study sites, with relatively high pH and many intermediate to rich fen species (*Sphagnum obtusum*, *S*. *subsecundum*, *Eriphorum gracile*, *Trichophorum alpinum*, *Sarmertypnum procerum*). This site had experienced one of the most significant expansions of a *Sphagnum*‐dominated zone with *S*. *fallax* as the main species of the transition zone, and bog lawns had *S*. *medium* coll. and *S*. *papillosum* (Säippäsuo W, Figure 2). Rich fens are not safe from vegetation changes under warming (Hájek et al., 2022; Kolari et al., 2021; Küttim et al., 2019) and their development may deviate significantly from trajectories of weakly minerotrophic poor fens.

It is well‐known that alternative trajectories towards raised‐bog development exist. Hughes and Barber (2004) recognized contrasting pathways of FBT either through dry or wet phases. In the dry scenario, a shift from fen vegetation to an intermediate stage occurs often with *Eriophorum vaginatum*‐dominated poor‐fen vegetation, indicating lowered or unstable water‐table level. The decay‐resistant fibers of *E*. *vaginatum* may facilitate hummock formation and further development of poor fens to bogs. In paleoecological research, *E*. *vaginatum* and *Sphagnum magellanicum* have been frequently connected to transitional stages throughout the Holocene (Hughes & Barber, 2003; Hughes & Dumayne‐Peaty, 2002; Tolonen, 1967; Tuittila et al., 2007; Väliranta et al., 2017). In our case, the succession of aapa mires followed the wet scenario of Hughes and Barber (2004), in which a sedge‐fen develops directly to a raised‐bog lawn, maintaining a high water‐table level throughout the FBT. Our transitional flark communities were mainly quaking *Sphagnum majus* and *S*. *jensenii* carpets, and water‐table levels had remained high. These species declined towards bogs where *S*. *balticum* became dominant in the more firmly structured lawns. The observed succession pattern can be considered as a type of secondary hydroseral development from a shallow water body to continuous mats of mosses fixed by vascular plant roots, and finally to a bog phase with peat accumulating above the groundwater level (Moore & Bellamy, 1974). This succession pattern has been reported less often from peat profiles (Granlund et al., 2022; Svensson, 1988; Turunen et al., 2002), but similar vegetation development has been found in case of studies by combining remote sensing and field observations (Kolari et al., 2022; Tahvanainen, 2011) and verified as recent changes (post‐LIA) by peat stratigraphies (including five out of our 23 study sites) with a high rate of lateral expansion of wet Sphagna, approximately 70 cm per year (Granlund et al., 2022). Similar to our spatial chronosequence approach, Svensson (1988) collected peat cores along a transect from a mud‐bottom hollow and *S*. *cuspidatum* carpet to *S*. *magellanicum* lawn and *S*. *fuscum—S*. *rubellum* bog in a southern Swedish mire, and correspondingly found *S*. *Cuspidata* peat above fen peat, topped by *S*. *magellanicum* peat.

The layers of *S*. sect. *Cuspidata* previously found in bog peat stratigraphies have been associated with a general rise in humidity and lake water levels (Svensson, 1988), and permafrost thaw in arctic regions (Magnan et al., 2018). In boreal aapa mire complexes, post‐LIA and recent warming and longer growing seasons have likely promoted the growth and expansion of *Sphagnum* mosses in wet flarks (Bengtsson et al., 2021; Granlund et al., 2022; Loisel et al., 2012). The moisture supply itself is rarely limited in undrained aapa mires that are fed by high snowmelt and groundwater recharge, enabling *Sphagnum* to take advantage of warming, although this might change in future (Heikkinen et al., 2022; Sallinen et al., 2023). But most importantly, the weakly minerotrophic conditions have allowed the presence of *S*. sect *Cuspidata* species in the fen flarks, thus, enabling their increase without delay from dispersal limitations. pH and concentrations of Ca and Mg were generally extremely low in the studied mire complexes, as pH ranged from 3.3 to 4.7 (with one exception of pH of 5.5), and both Ca and Mg concentrations were under 1 mg/L in the fen flarks. As expected, the pH and concentrations of Ca and Mg were higher in the fen flarks than in transitional communities and bogs. Groundwater pH and Ca concentration are relatively low in the whole Fennoscandia due to glacial history and calcium‐poor bedrock (Hájek et al., 2021), and boreal fens with weak minerotrophy and poor mineral buffering capacity (Tahvanainen et al., 2002) are sensitive to acidification, which may increase susceptibility to *Sphagnum* increase and FBT (Tahvanainen, 2011). Once established, *Sphagnum* mosses can further lower pH by the production of organic acids and cation exchange (Clymo, 1963, 1964; Schweiger & Beierkuhnlein, 2017). In our study sites, DOC concentration was slightly but not significantly elevated in the transition zones, while in bogs DOC was significantly higher than in the fens and transition zones. Increased DOC concentrations likely connect to the lowered pH, as organic acids are mainly responsible for acidity in mire waters (Tahvanainen et al., 2002), but more detailed water chemical analyses and monitoring would be needed to assess the role of water chemistry.

### 4.3 Diversity patterns along the fen‐bog chronosequence

The string‐flark patterning is an important part of diversity in aapa mires. Flarks often have sparse vegetation cover, with few specialized species like the carnivorous *Utricularia* spp. and some aquatic mosses, but high species richness in strings increases diversity. In our study sites, species richness was generally higher in bogs than in fen and transitional communities, the only exception being relatively low richness in bryophytes in bog hummocks. It must be noted that a majority of our study sites were weakly minerotrophic and some had open‐water pools, thus; the flark fens did not harbor rich fen vegetation. At sites with higher minerotrophy and rich fen vegetation, the pattern of species richness would likely be the opposite, decreasing towards bogs. In any case, our results suggest that the progressive fen‐to‐bog succession will likely lead to lowered compositional heterogeneity of plant communities. This is indicated firstly by the significantly higher beta diversity in fens compared with bogs, and secondly by the greater variation in species composition among fen than bog sites, as indicated by the spread in NMDS ordinations and comparison of within‐zone mean Bray–Curtis dissimilarities.

Aapa mires were assessed as Least Concern in the European Red List of Habitats (Janssen et al., 2016), but the assessment was lacking estimation based on quantitative criteria and potential changes along the FBT trajectory could not be assessed. In the Red List of Finnish habitats, southern aapa mire complex types were assessed as near threatened to threatened, mainly affected by the loss of area and quality by ditching (Kontula & Raunio, 2019). Habitat changes due to natural succession in response to climate change are difficult to assess, as recognition of key mechanisms is demanded, and reliable studies are needed to infer the scale of potential changes. The overgrowth of acidophilic *Sphagnum* mosses reported here and in several case studies (Granlund et al., 2022; Kolari et al., 2022; Tahvanainen, 2011) can be a common phenomenon that clearly poses a threat to aapa mire habitat types, but studies so far have focused on exploring the phenomenon, while representative data is lacking on what share of aapa mires is affected. The mosaic pattern of flarks and strings, and the aquatic primary production in flarks are important also for other taxonomical groups than plants. Flarks provide feeding grounds and hummock strings nesting places for birds, including many waders. Thus, the flark infilling and FBT may pose a threat to populations of bird species of mire habitats, that have already declined since the end of the 20th century (Fraixedas et al., 2017; Virkkala & Rajasärkkä, 2012).

### 4.4 Transitional plant communities in the context of climate change and ecosystem carbon balance

Under the current circumstances of global climate change, the fate of mire carbon storage has received particular attention. Most studies have focused on the direct effects of warming‐related treatments on greenhouse gas fluxes, while the indirect effects of plant community changes have often been neglected. However, the effect of warming on net ecosystem CO_2_ exchange depends on vegetation composition, and the efflux of CH_4_ is more strongly controlled by plant communities than by temperature (Ward et al., 2013). Additionally, the effects of climate change on mire vegetation are likely different in different types of fens and bogs (Kokkonen et al., 2019). While *S*. sect. *Cuspidata* can increase in aapa mire complexes with sufficient water supply (Granlund et al., 2022; Kolari et al., 2022) and species of low hummocks in rich fens (Kolari et al., 2021), prolonged warming and possible lengthening of draught periods may, however, cause desiccation damage and decreased carbon uptake, as inferred for bog Sphagna (Nijp et al., 2014; Norby et al., 2019). Supporting our results of progressive succession instead of desiccation damage, modeling results have indicated increasing carbon sequestration in northern peatlands with increased productivity due to lengthening growing season in the near future (Gallego‐Sala et al., 2018), as also indicated by patterns of carbon accumulation in peatlands during the last millennium (Charman et al., 2013). Our results indicate that vegetation changes can contribute in the same direction or act as a mechanism to increase productivity. *Sphagnum* mosses are known to readily benefit from longer growing seasons (Loisel et al., 2012), as they can continue growing in warm autumns, while many vascular plants are constrained by growth phenology.

Increase of transitional *S*. sect. *Cuspidata—Scheuchzeria palustris*‐type vegetation over fen flarks leads to increased accumulation of decay‐resistant *Sphagnum* peat and soil carbon (Granlund et al., 2022; Loisel & Yu, 2013). An increase in *Sphagnum* mosses can also reduce CH_4_ emissions, particularly in rich fens (Zhang et al., 2021), thus, having climate cooling feedback. However, many vascular plants contribute to mire ecosystem carbon cycle by transporting CH_4_ from deep anoxic peat to the atmosphere through plant aerenchyma tissues (Lai, 2009), and an increase in aerenchymatous sedges can enhance CH_4_ flux in wet flarks (Strack et al., 2006). In our study, the cover of deep‐rooted aerenchymatous species did not differ between fen, transitional, and bog communities, although it was significantly higher in strings and bog hummocks than in flarks and bog lawns. The cover of shallow‐rooted aerenchymatous species was significantly higher in transitional flarks compared with fen flarks and bog lawns, which resulted mainly from the increase in *Scheuchzeria palustris*. Dorodnikov et al. (2011) found that the rate of transported CH_4_ from peat to the atmosphere was higher in hollows with *S*. *palustris* when compared to drier lawns and hummocks characterized by *E*. *vaginatum*. In fact, *S*. *palustris* has among the highest methane transport rate of boreal fen species, and it can be responsible for almost half of the ecosystem‐scale plant transport in boreal fens (Korrensalo et al., 2022).

## 5 CONCLUSIONS

The identification of the decadal time scale chronosequence of succession from fen to bog made it possible to explore details of vegetation change and test hypothetical differences among functional species groups. Bryophyte cover and communities of flarks appeared most responsive to the FBT, while bryophytes in hummock strings and vascular plants, in general, had more overlap across the chronosequence communities. Our study highlights the potential of ongoing and future shifts in wet boreal fens to *Sphagnum*‐dominated bogs through a transitional phase with *Sphagnum* sect. *Cuspidata—Scheuchzeria palustris*‐type vegetation, while maintaining water‐saturated conditions. This leads to the infilling of fen flarks, and the development continues with the formation of bog lawns and hummocks. The transition phase is intermediate in species composition and water chemistry between fen and bog types, with few unique characteristics. In this trajectory, an increase in water‐table depth results from the vegetation succession, as the moss surface ascends higher, which may lead to increased accumulation of carbon in the progressively developing *Sphagnum* peat. Our results suggest that the shift from fen‐to‐bog vegetation reduces habitat heterogeneity of aapa mires and leads to more homogenous ombrotrophic bog vegetation. Fennoscandian mire complexes are often weakly buffered with low mineral concentrations, making them sensitive to acidification and ombrotrophication. Changes are likely driven by climate warming, as evidenced in recent studies but can also be triggered by hydrological alterations that call for restoration measures to conserve biodiversity. However, careful considerations are in place with restoration planning, as the bog expansion over aapa mires follows a natural succession pathway of mires, and it likely has desirable cooling feedback. Recognizing progressive responses to changing climate and differentiating from degradation is important and timely in northern mire ecosystems.

## AUTHOR CONTRIBUTIONS

**Tiina H. M. Kolari:** Conceptualization (equal); formal analysis (lead); funding acquisition (equal); investigation (lead); methodology (lead); visualization (lead); writing – original draft (lead). **Teemu Tahvanainen:** Conceptualization (equal); formal analysis (supporting); funding acquisition (equal); investigation (supporting); methodology (supporting); project administration (lead); supervision (lead); writing – review and editing (lead).

## CONFLICT OF INTEREST STATEMENT

We declare no conflict of interest.

## Supporting information


Appendix S1.

## Data Availability

Kolari, T. H. M. & Tahvanainen T. (2022). Plant species and water chemistry data for “Inference of future bog succession trajectory from spatial chronosequence of changing aapa mires” [Dataset]. Zenodo. https://doi.org/10.5281/zenodo.7319537
