# Supplementary material for: Inference of future bog succession trajectory from spatial chronosequence of changing aapa mires
Source: Ecol Evol. 2023 Apr 18;13(4):e9988. doi: 10.1002/ece3.9988 (PMC10111175; doi:10.1002/ece3.9988)

**Supplement 1**

Inference of future bog succession trajectory from spatial chronosequence of changing aapa mires. *Ecology and Evolution.*

Tiina H. M. Kolari^1^ & Teemu Tahvanainen^1^

¹Department of Environmental and Biological Sciences, University of Eastern Finland, P.O. Box 111, FI-80101, Joensuu, Finland

**Comparisons of historical aerial photographs (1944-1970) and new (2016-2021) color-infrared orthophotos for each site (in alphabetical order)**

All images are freely accessible photographs from the National Land Survey of Finland.


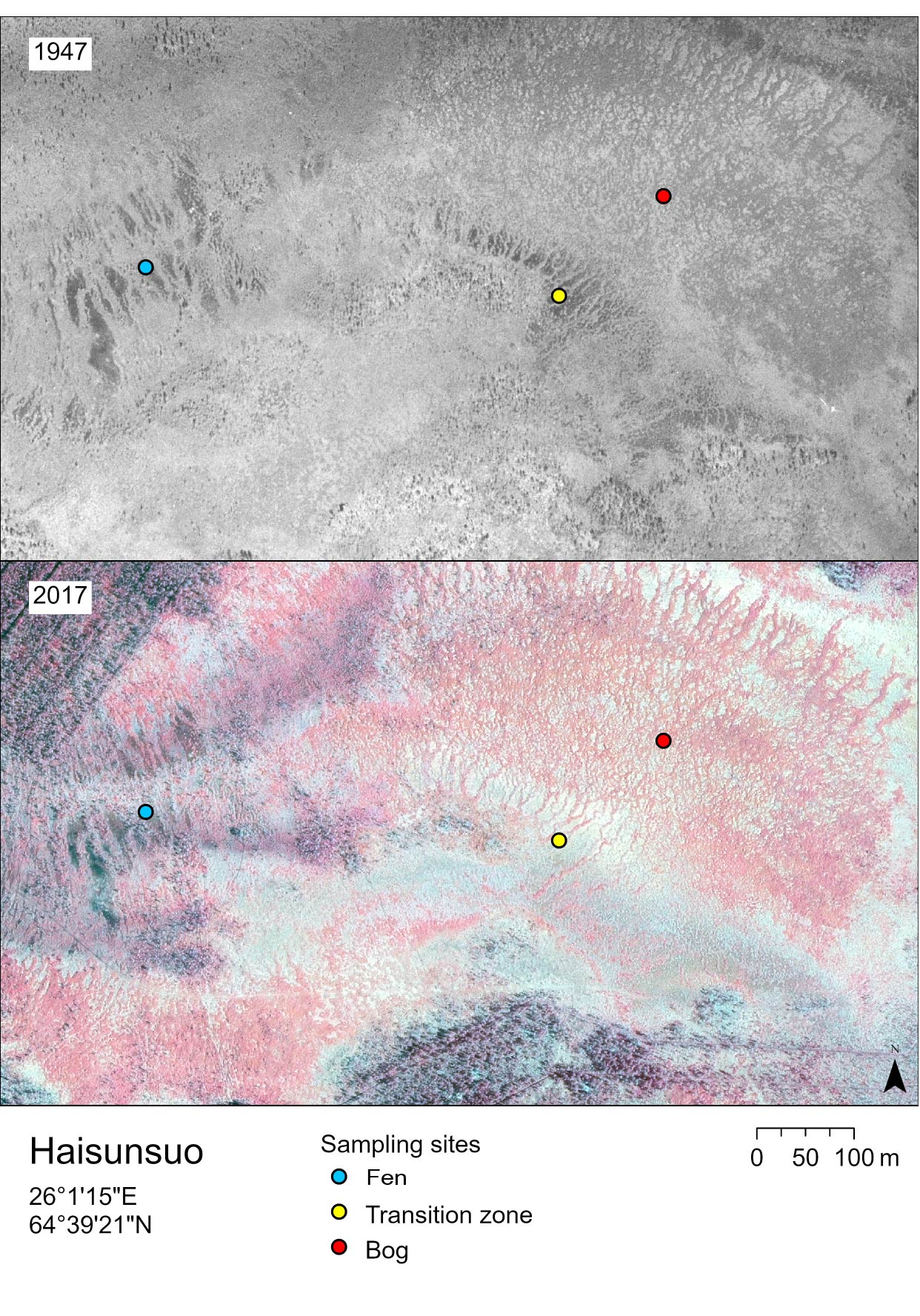


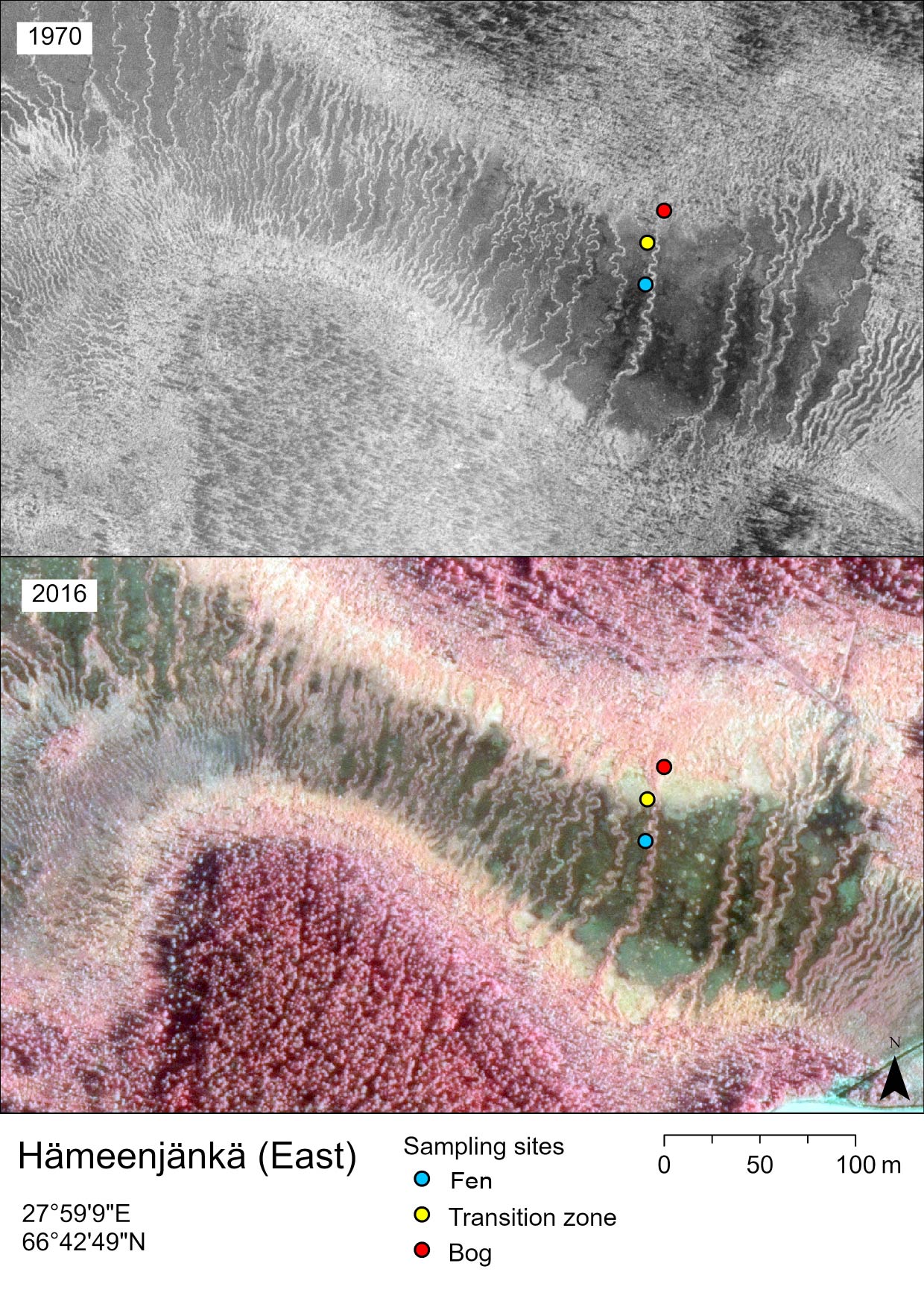


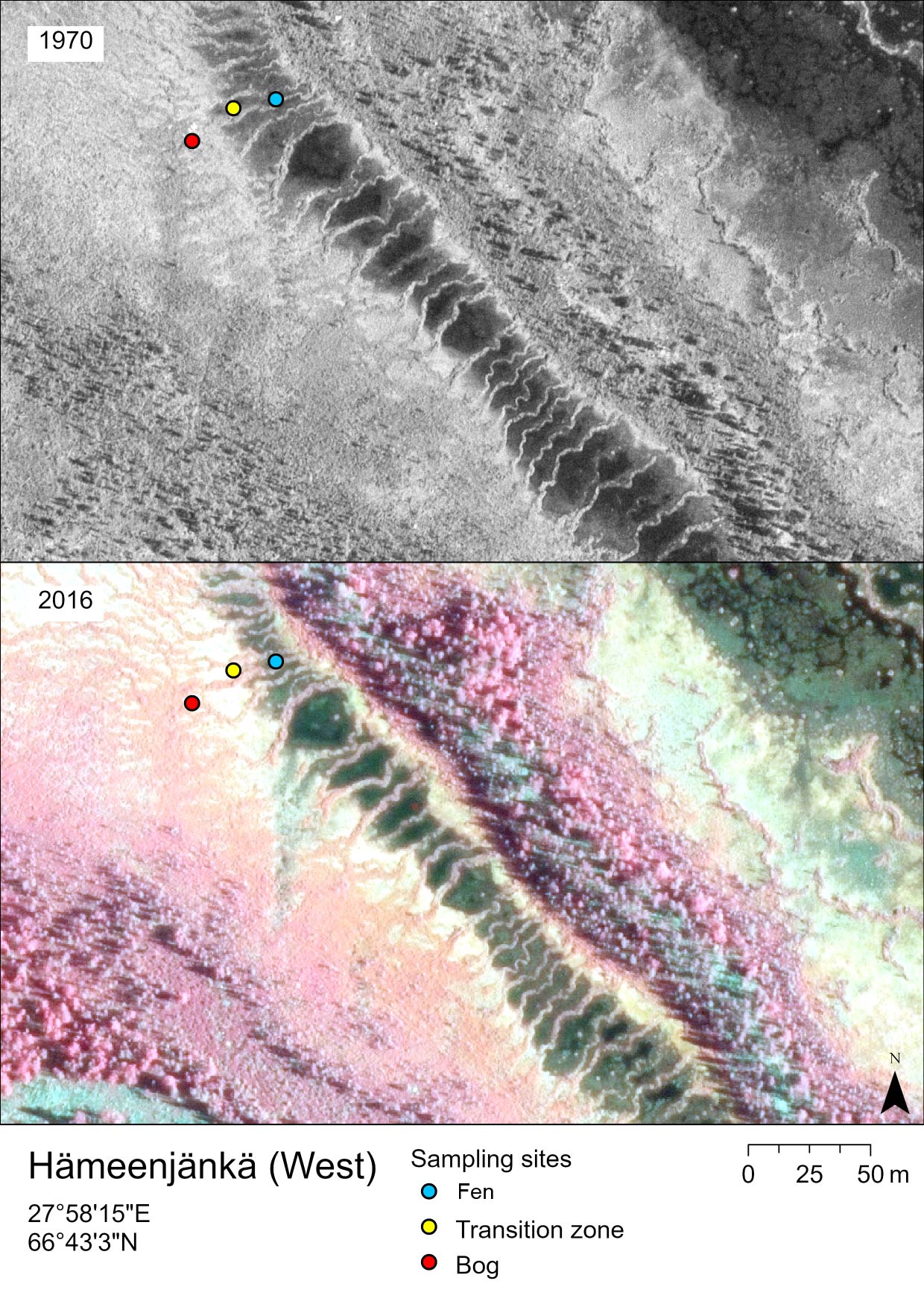


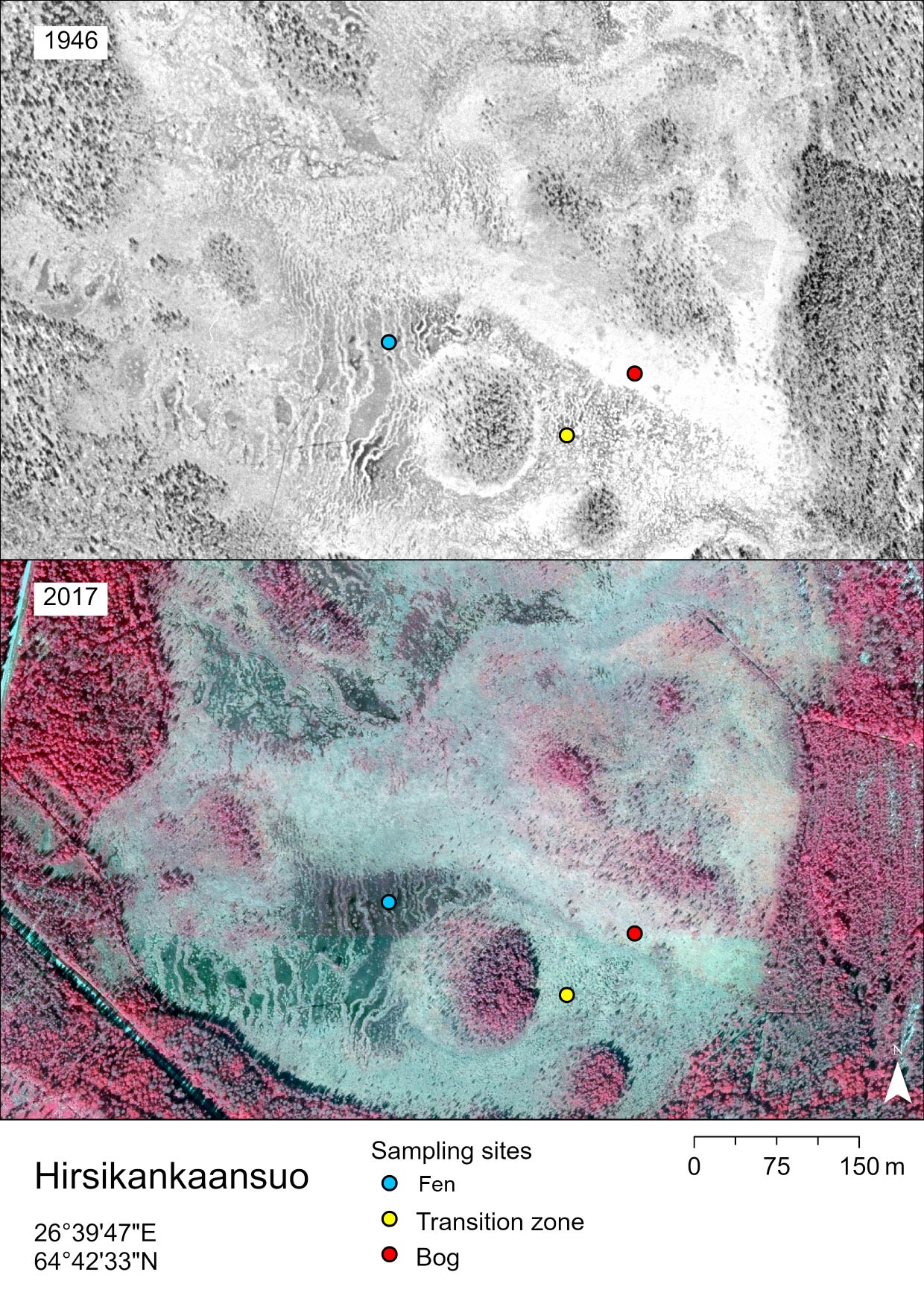


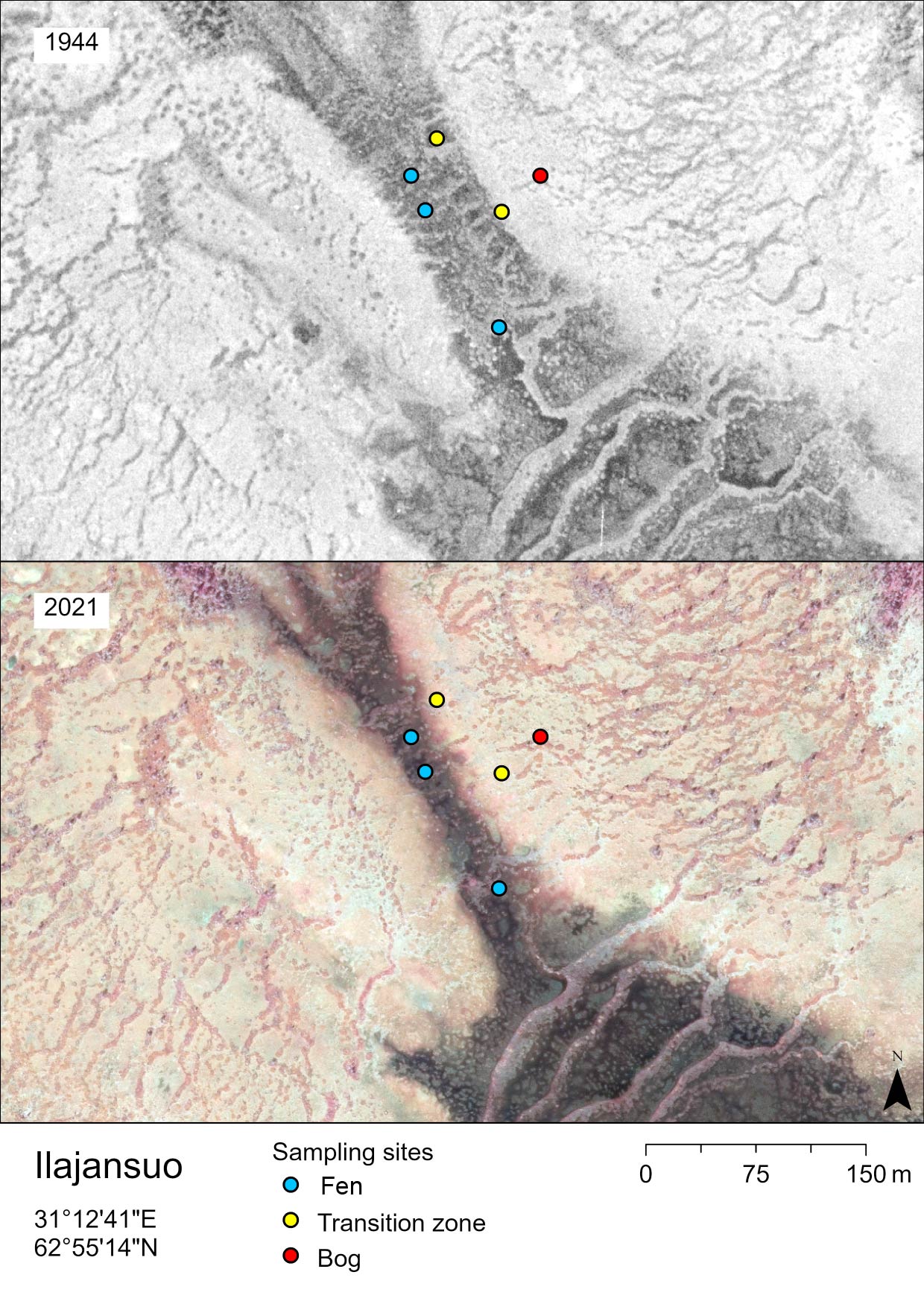


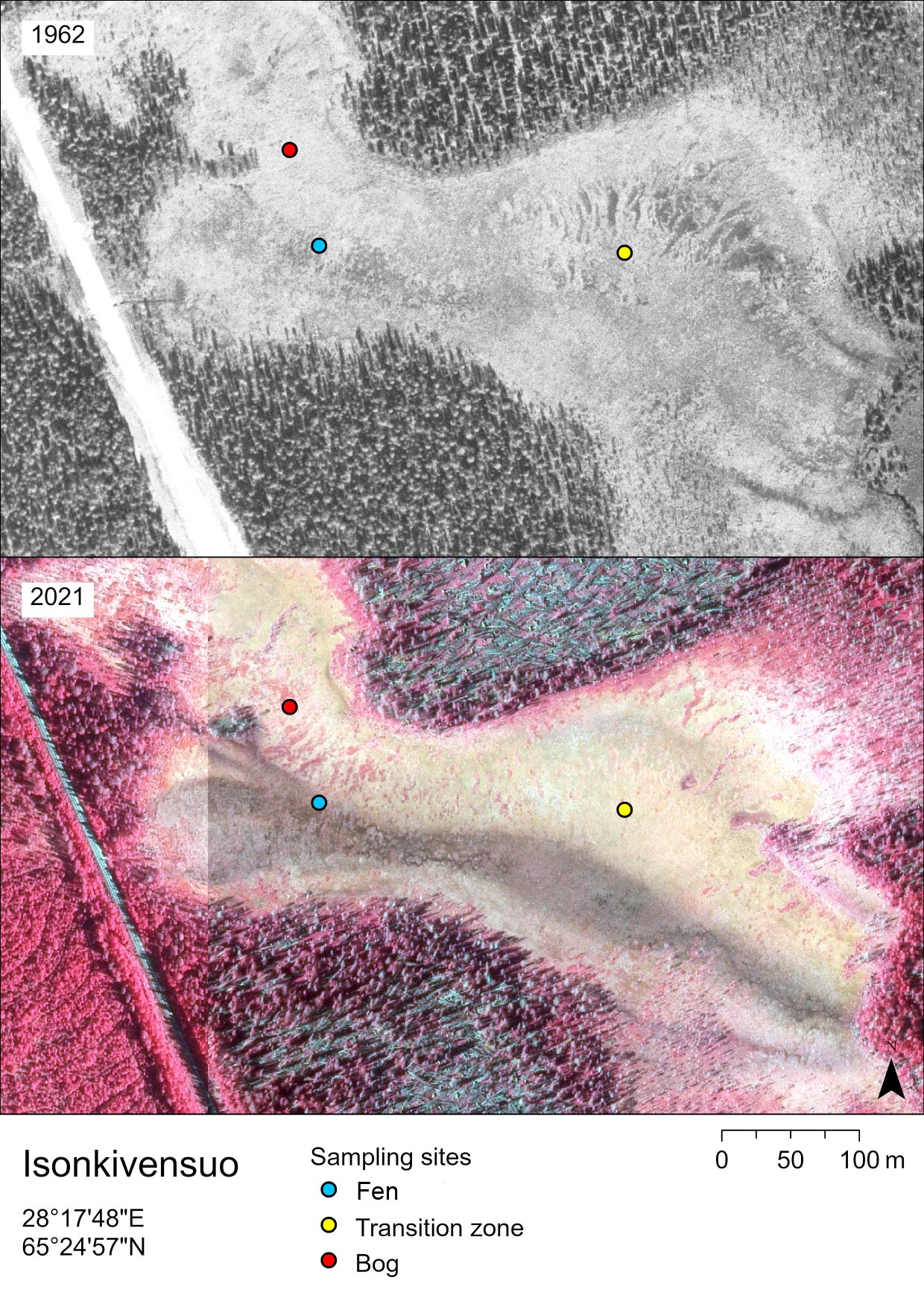


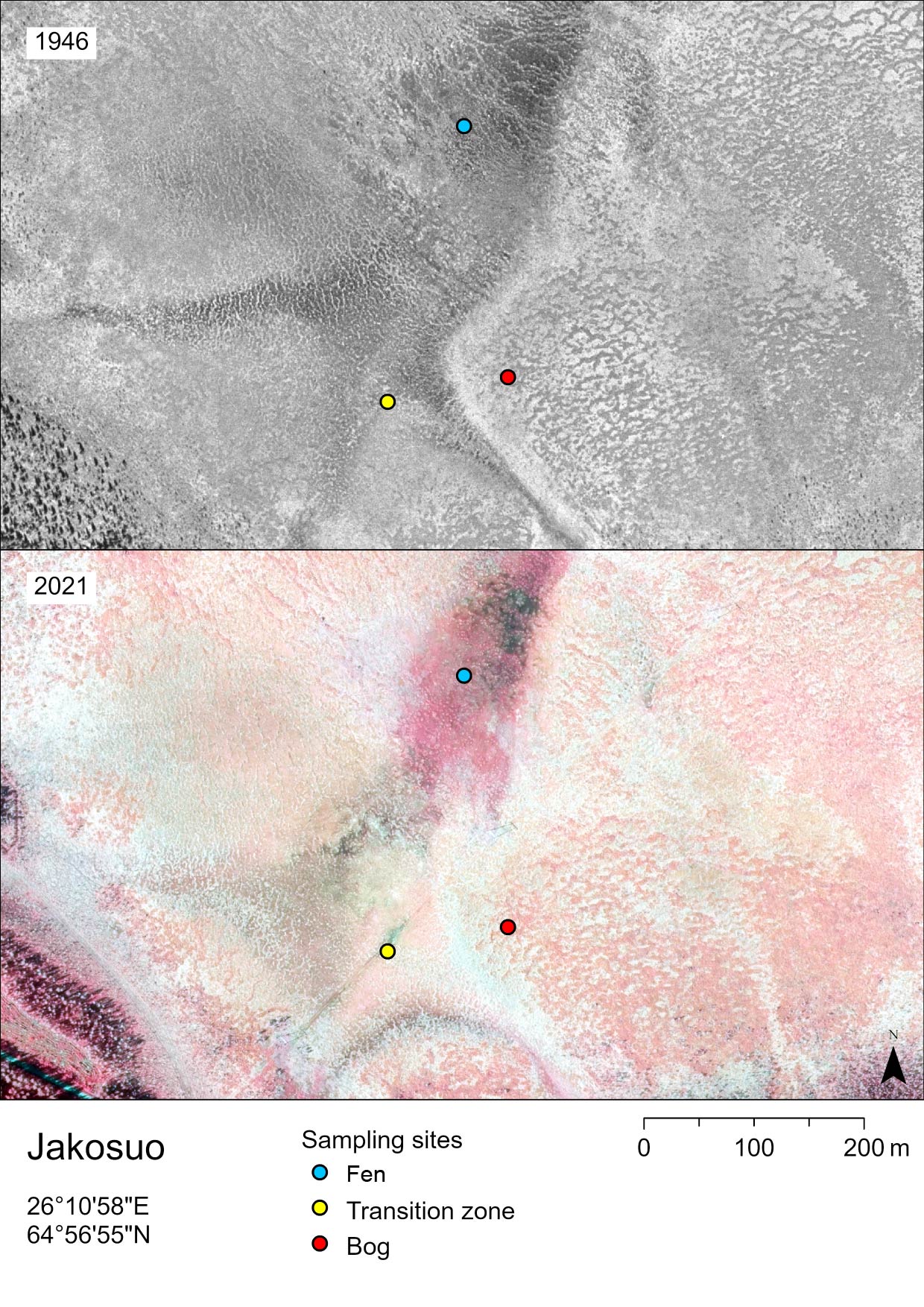


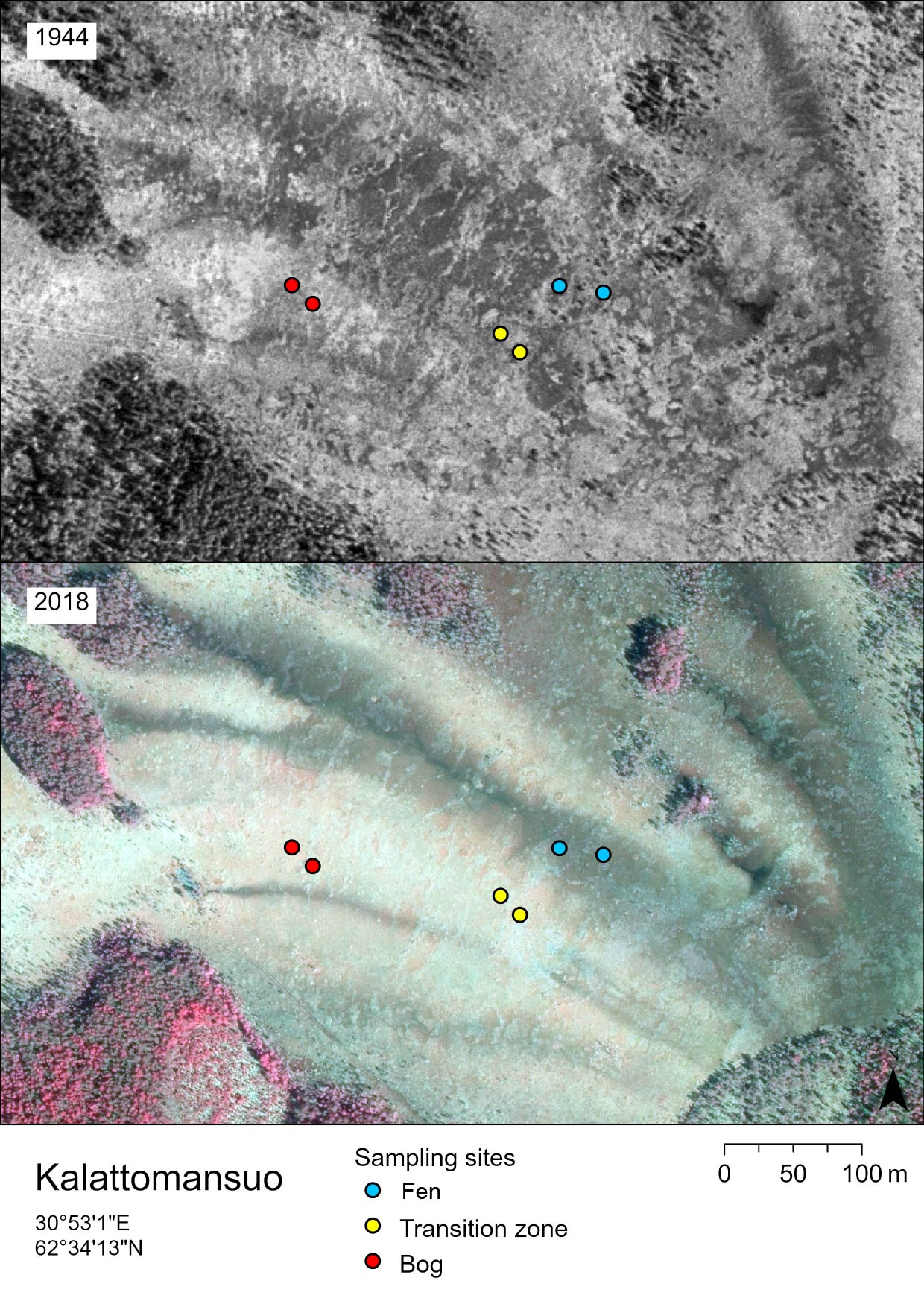


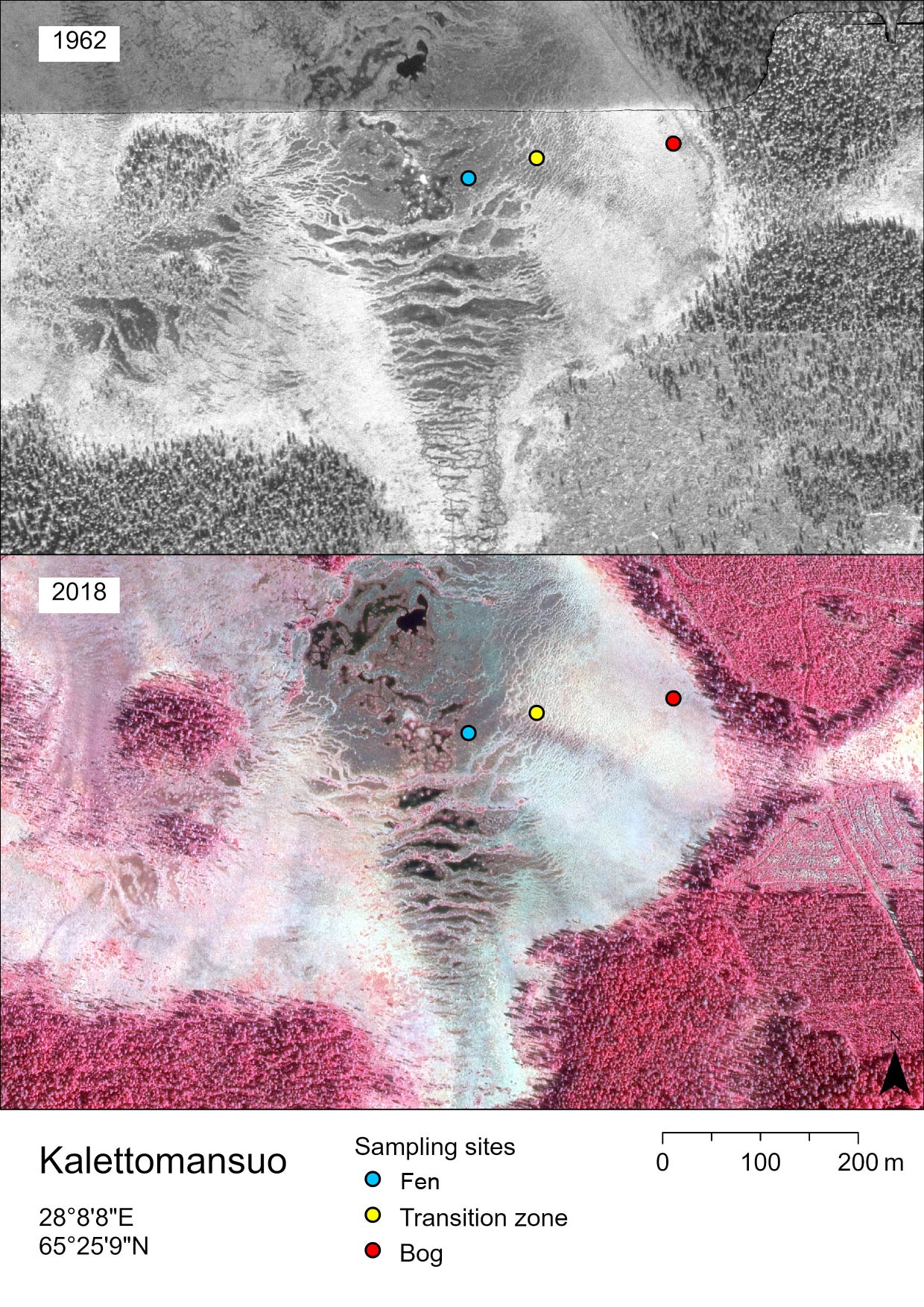


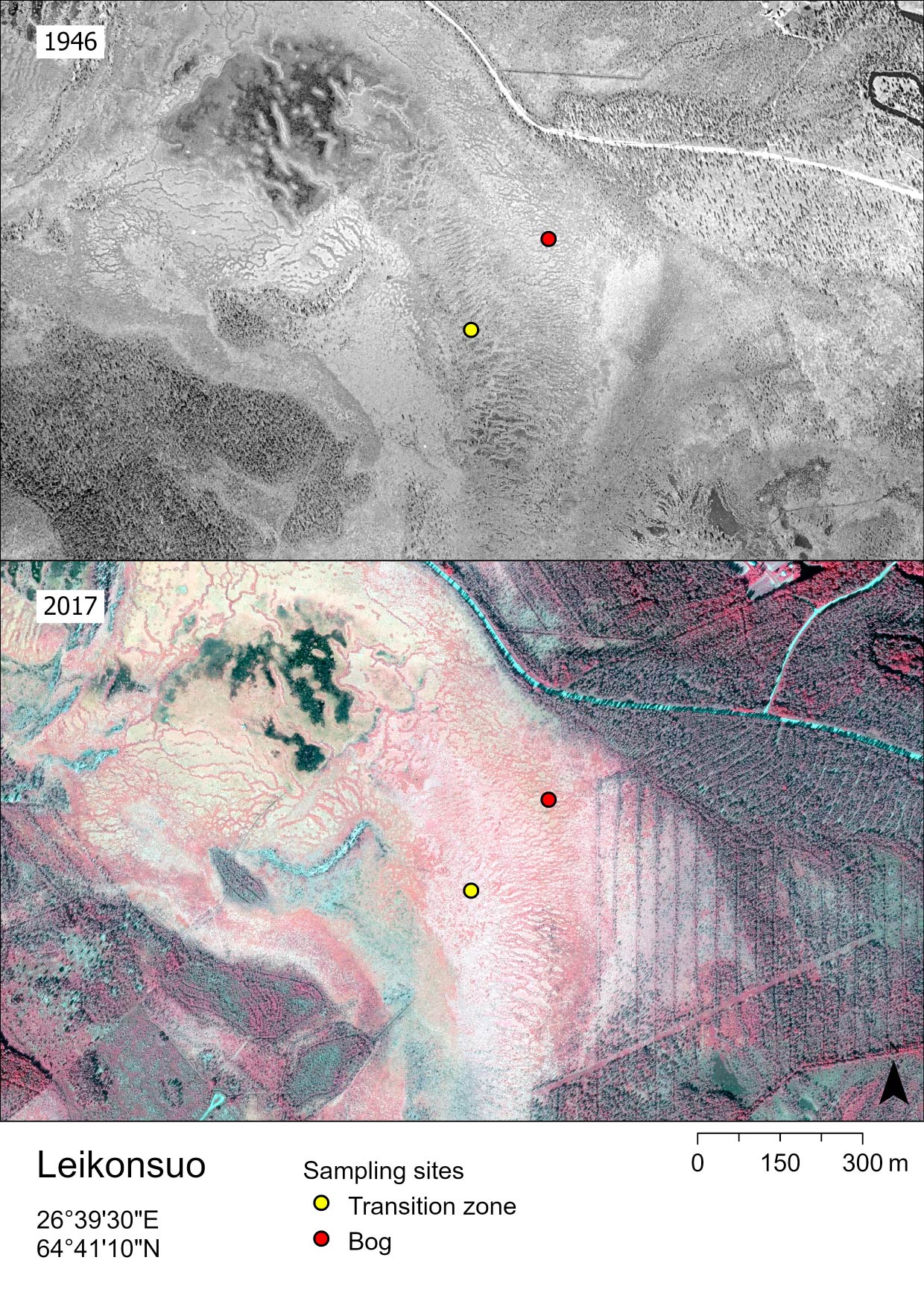


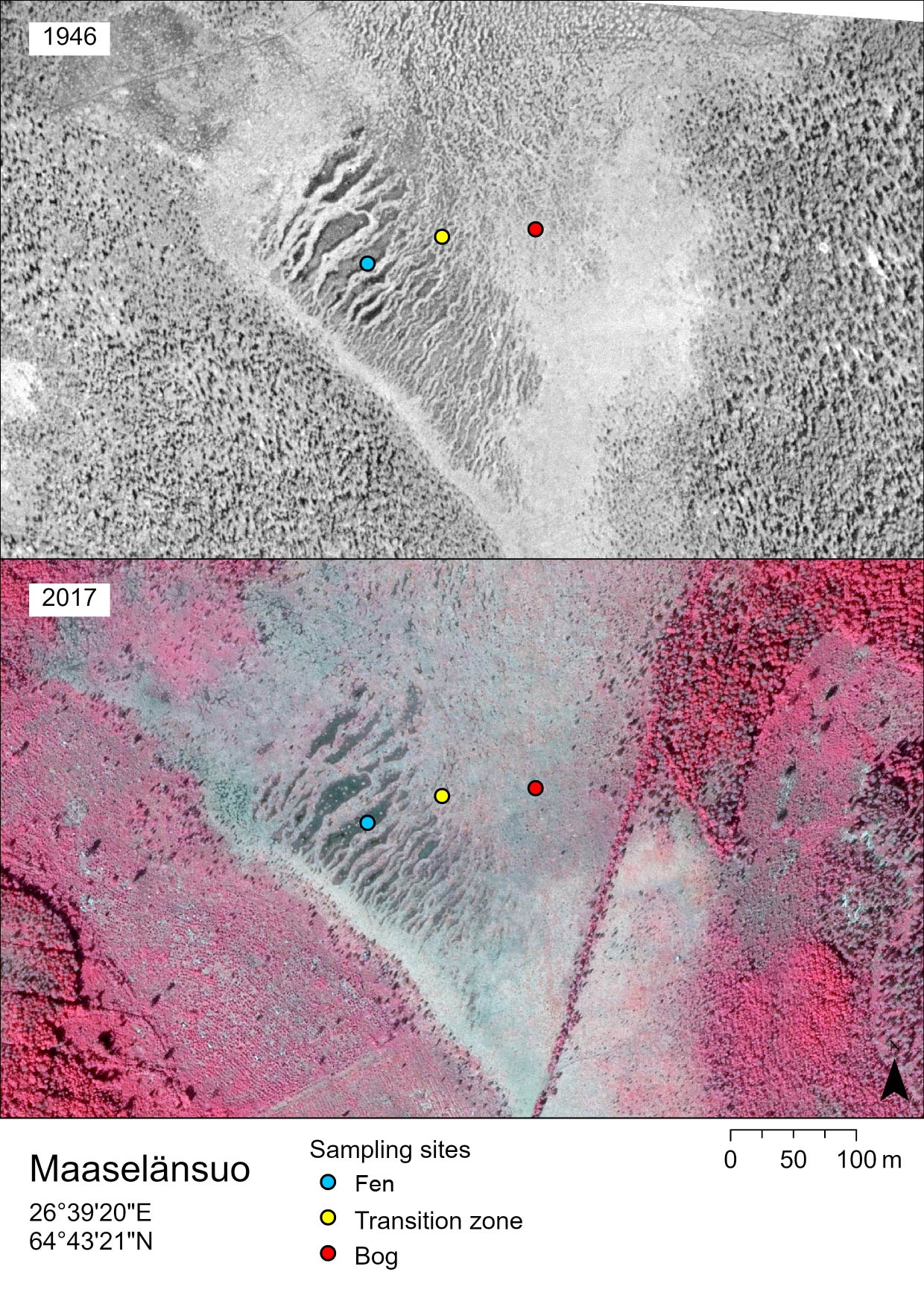


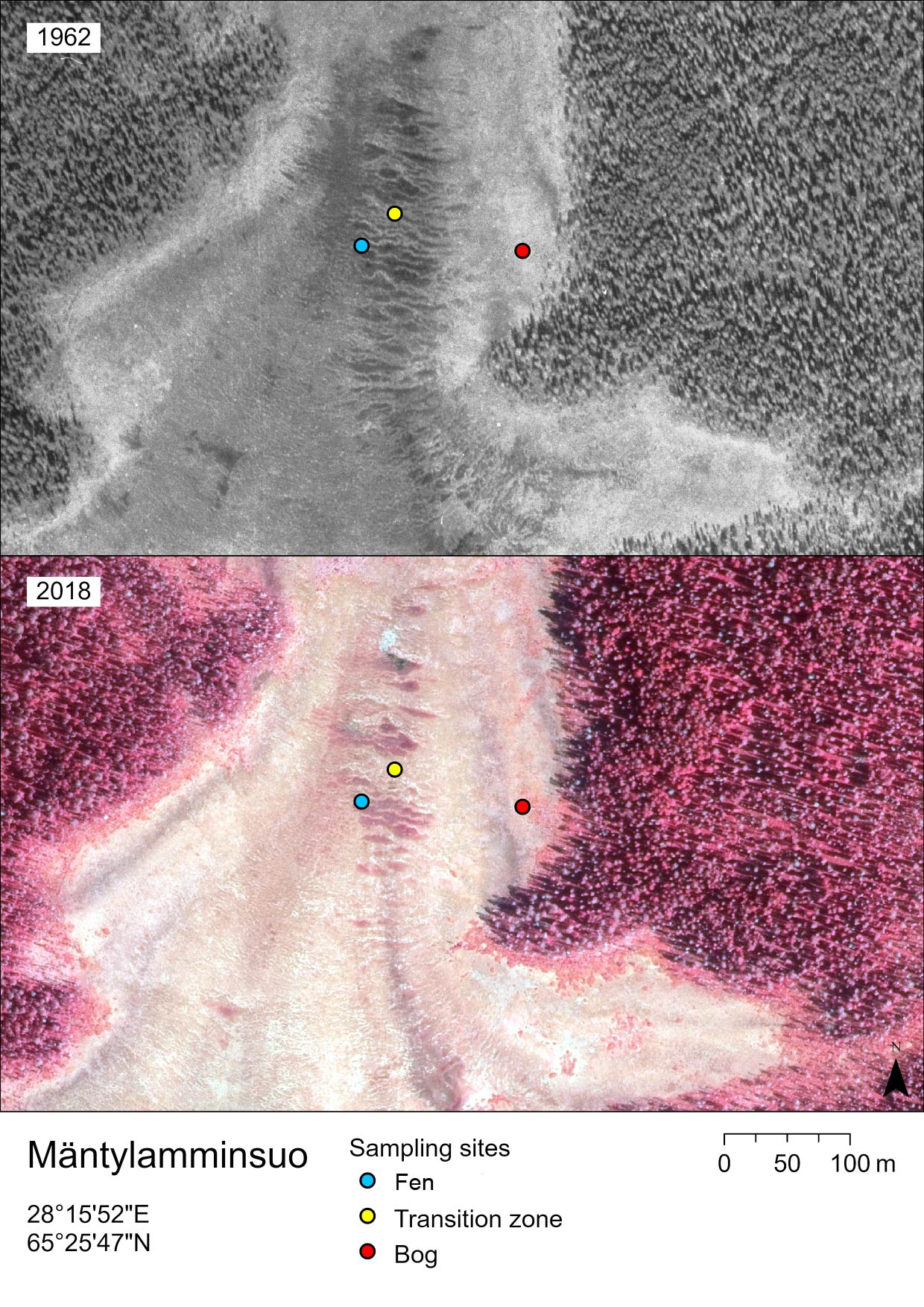


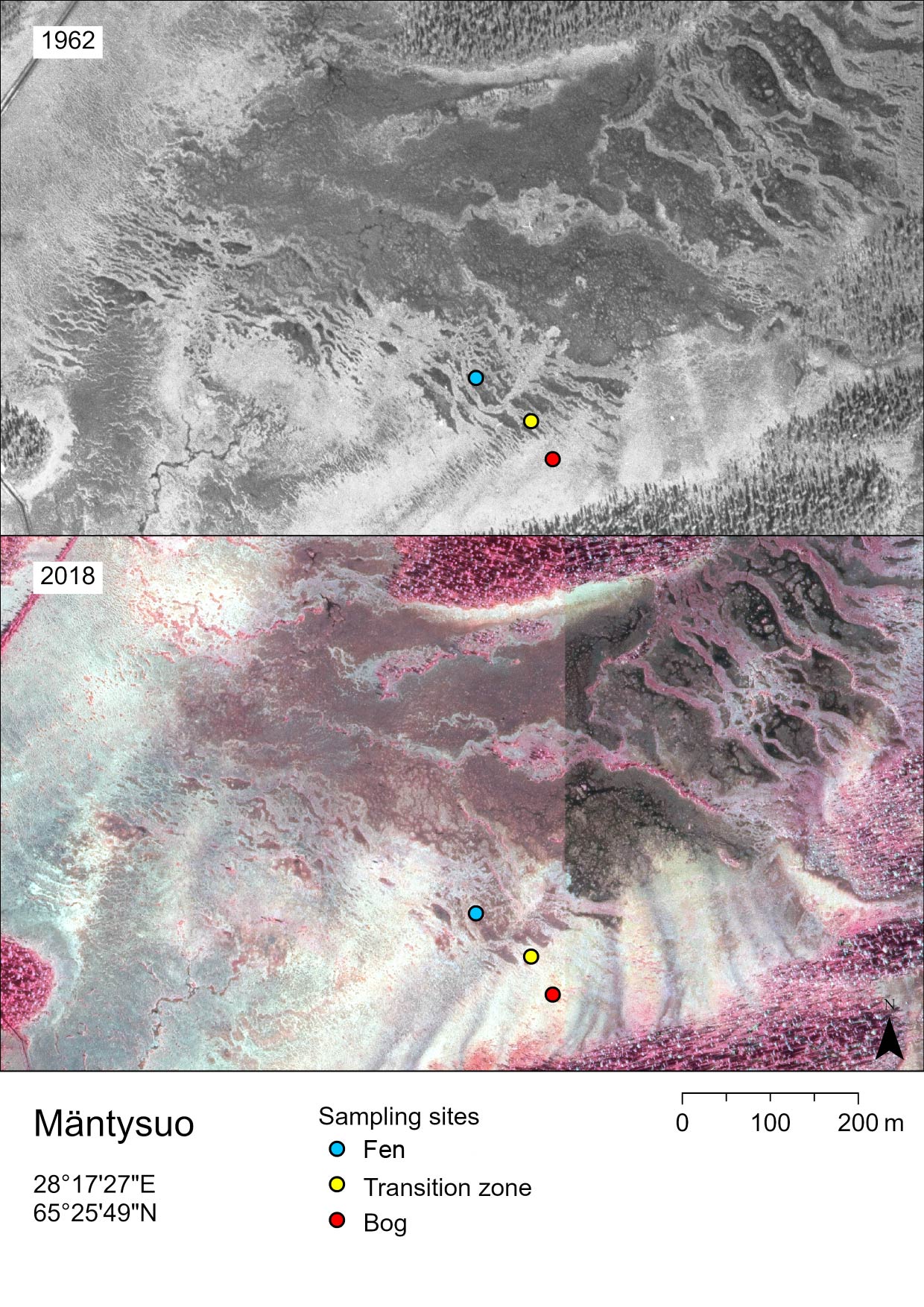


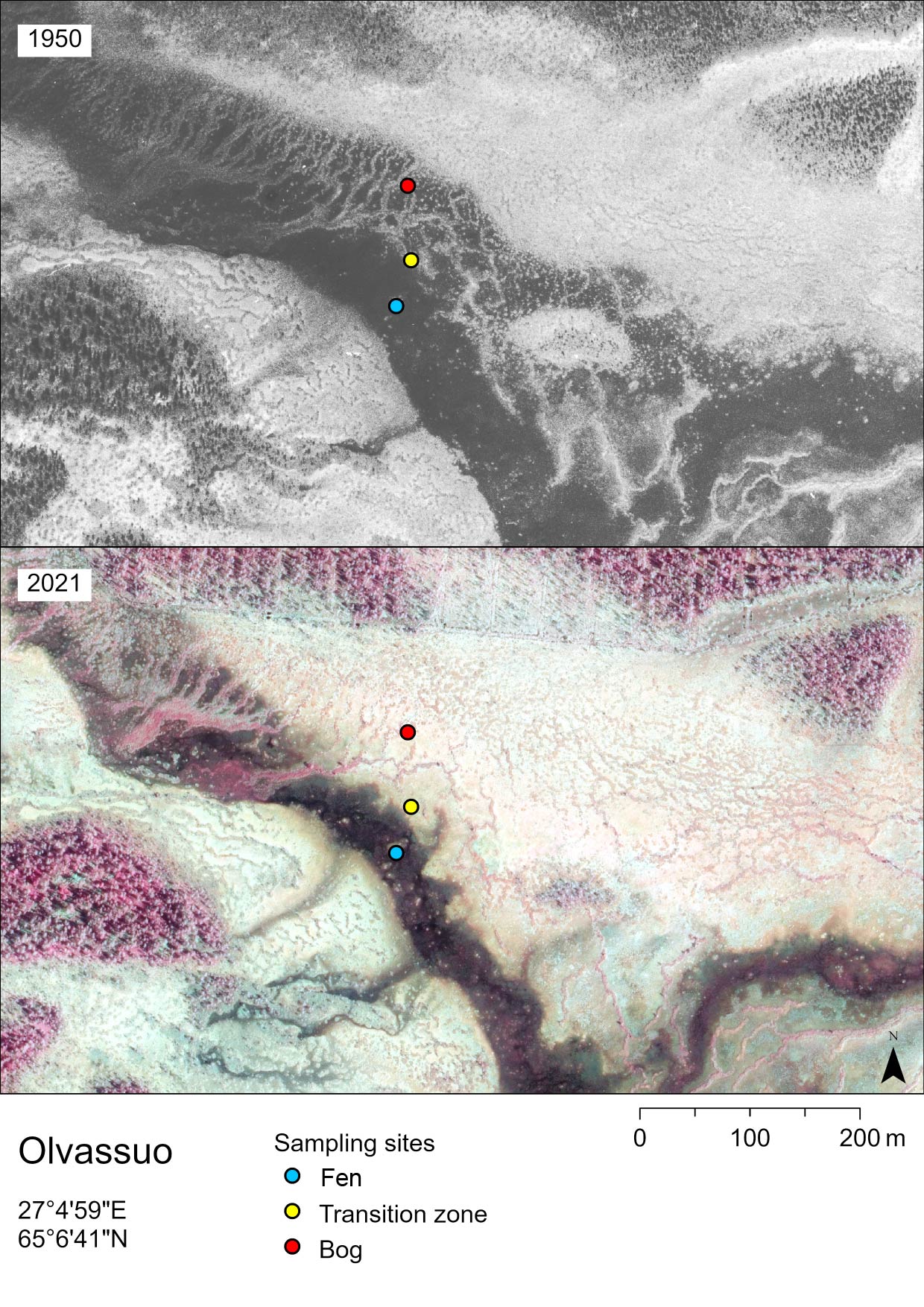


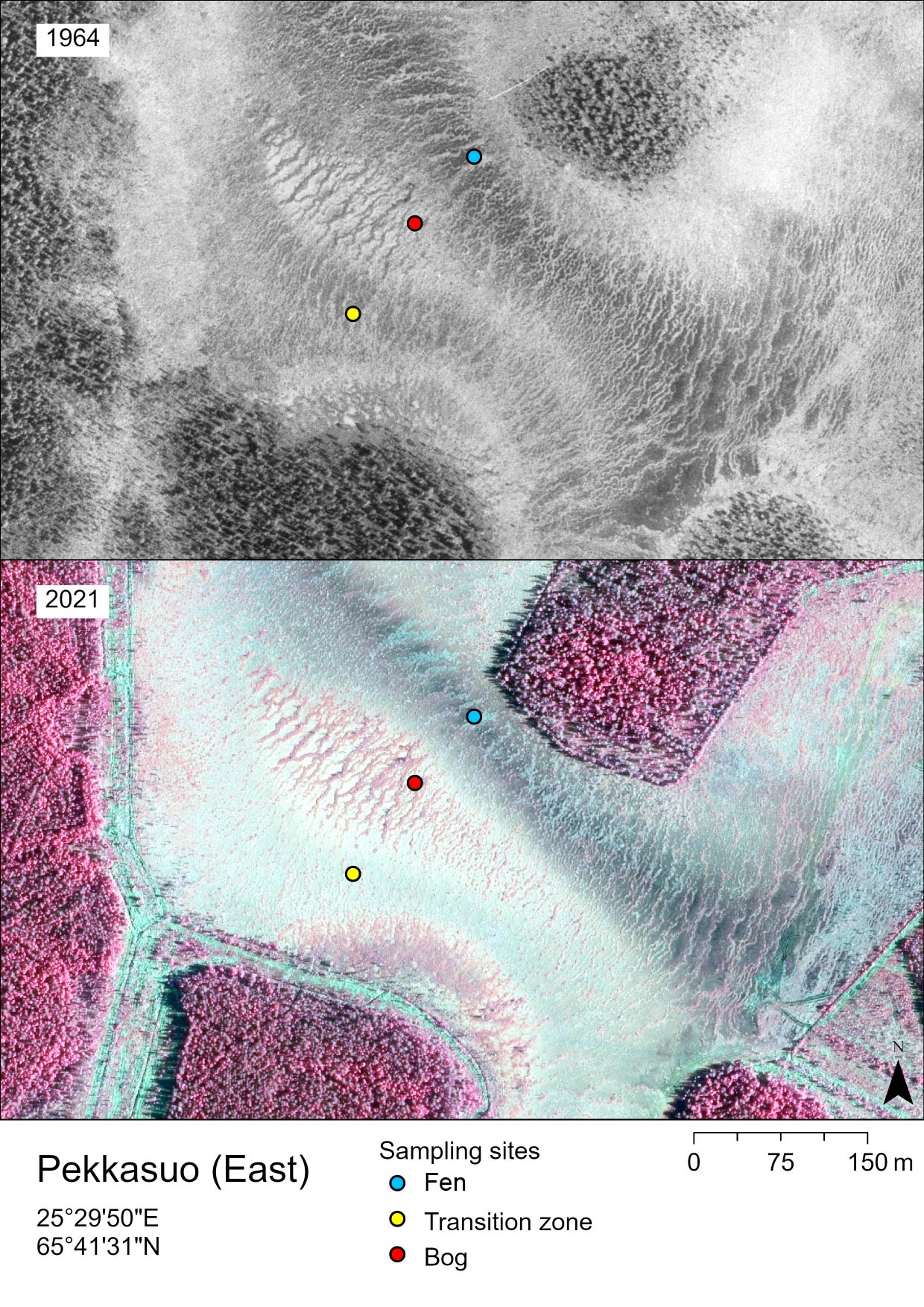


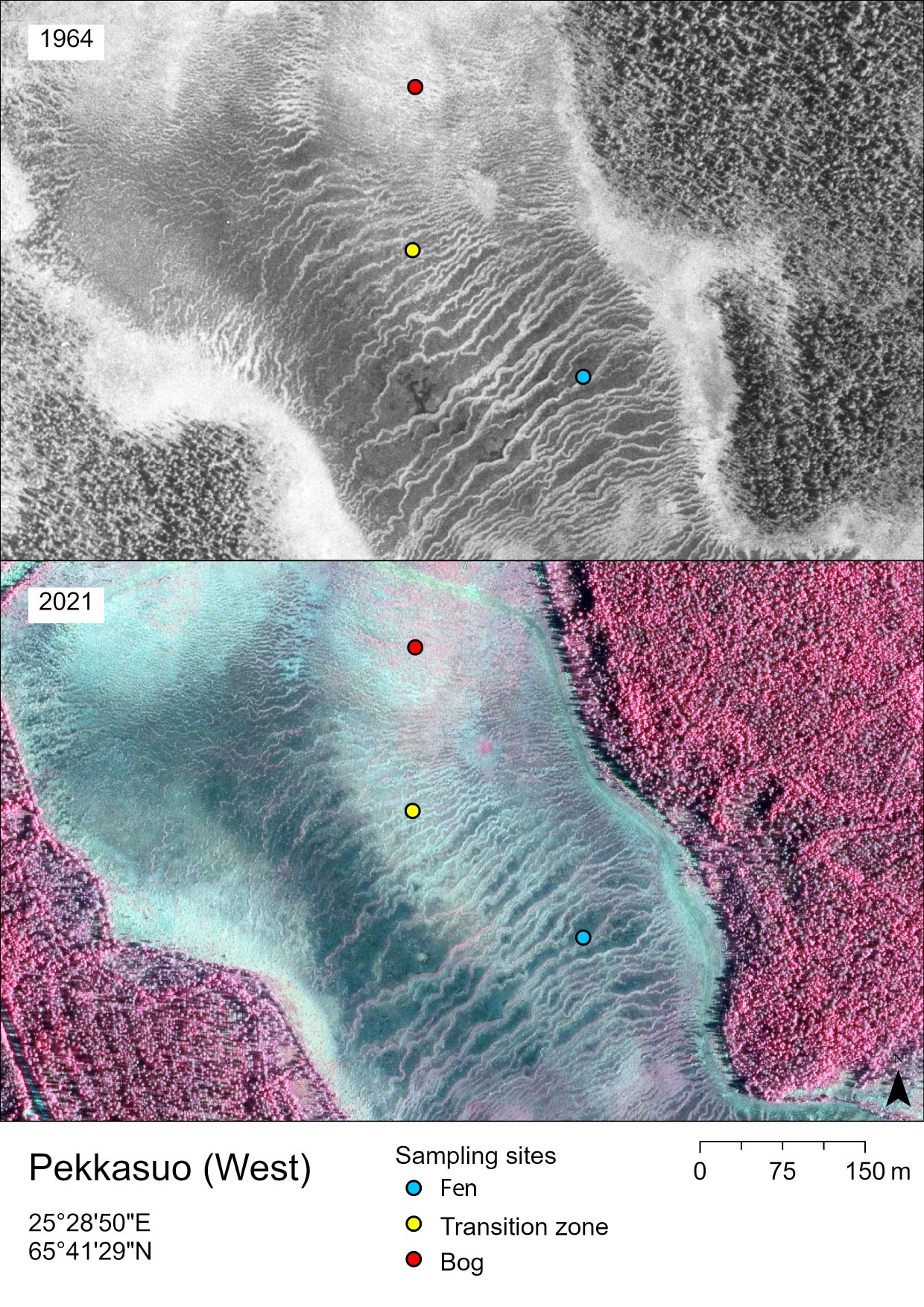


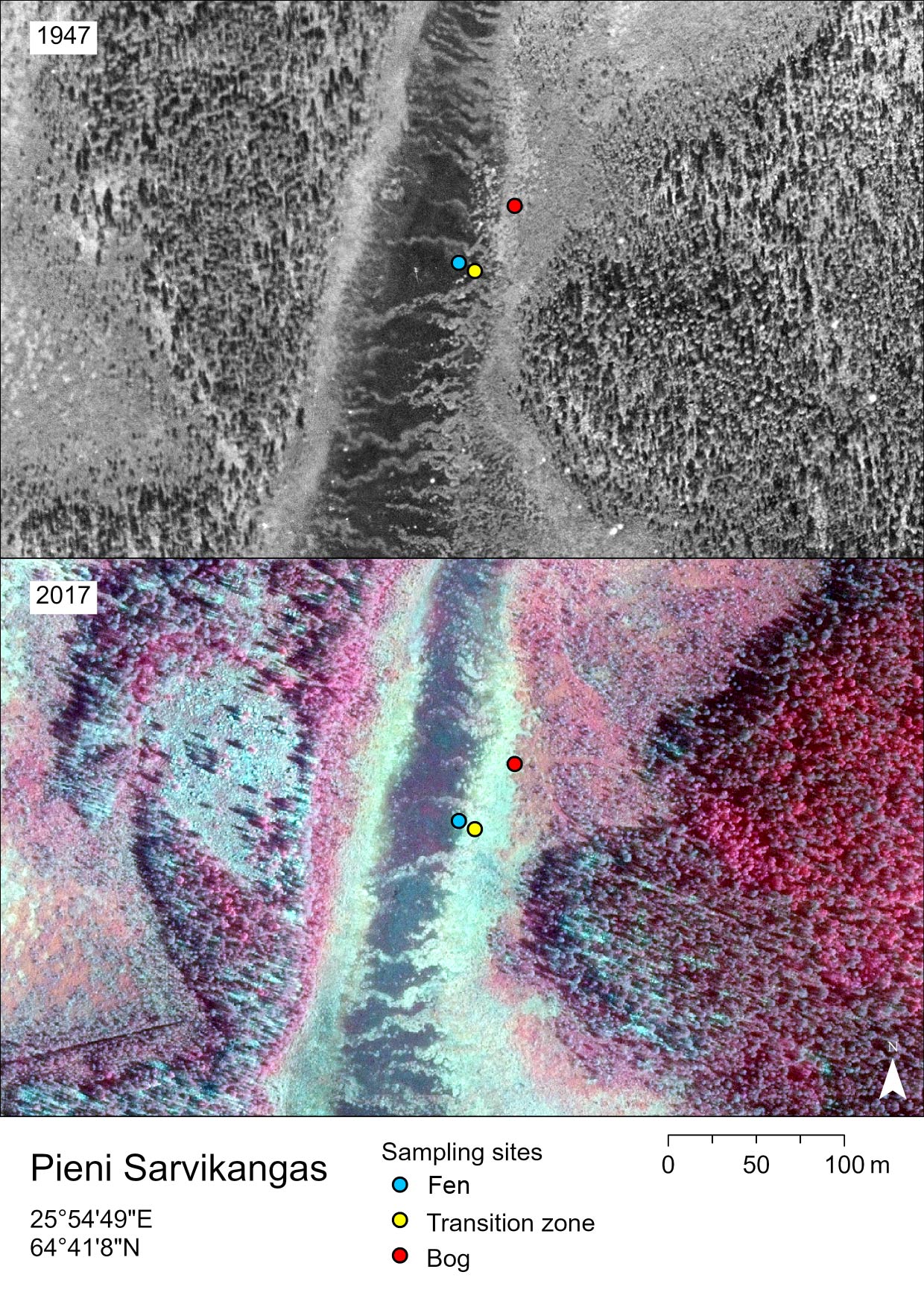


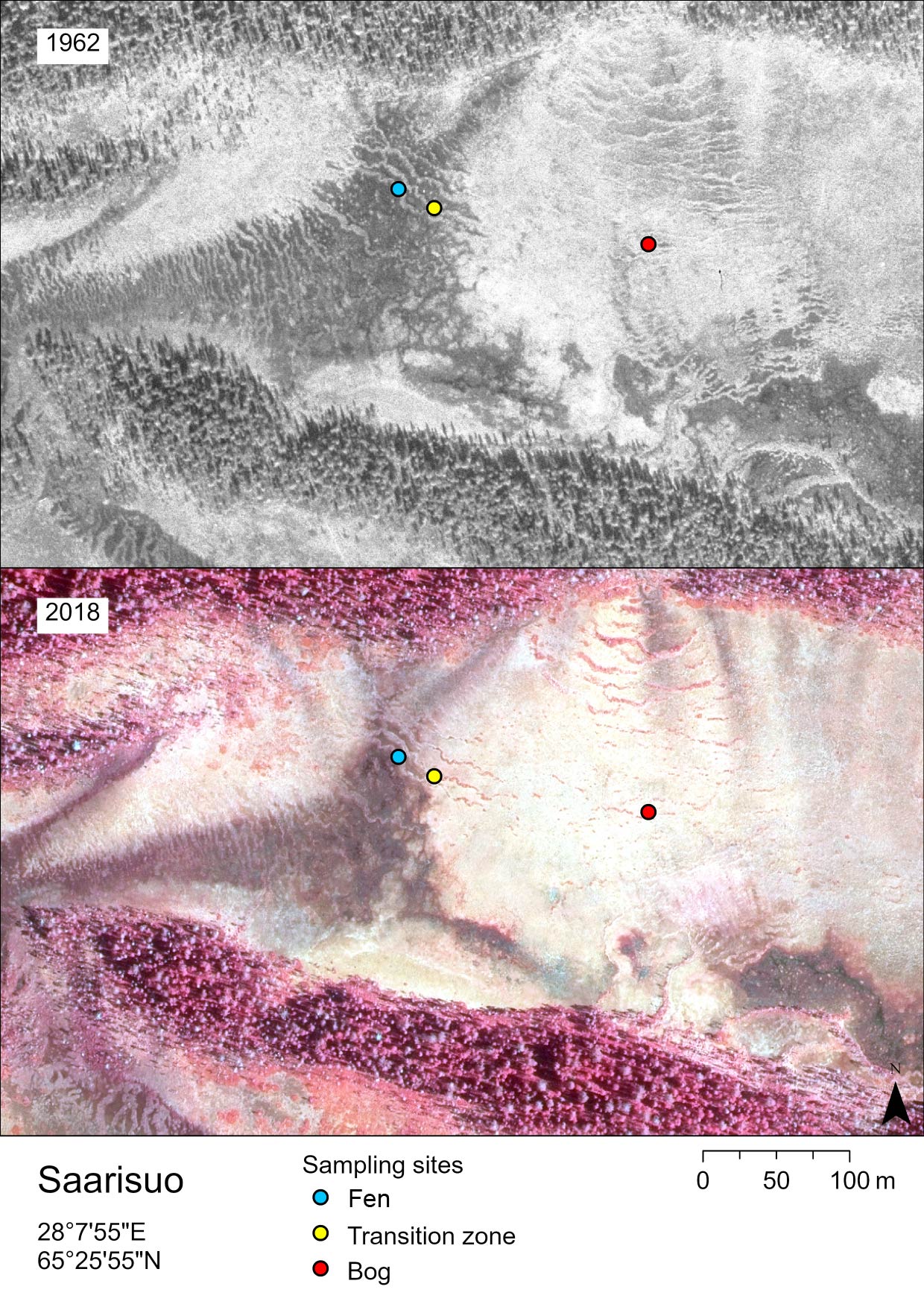


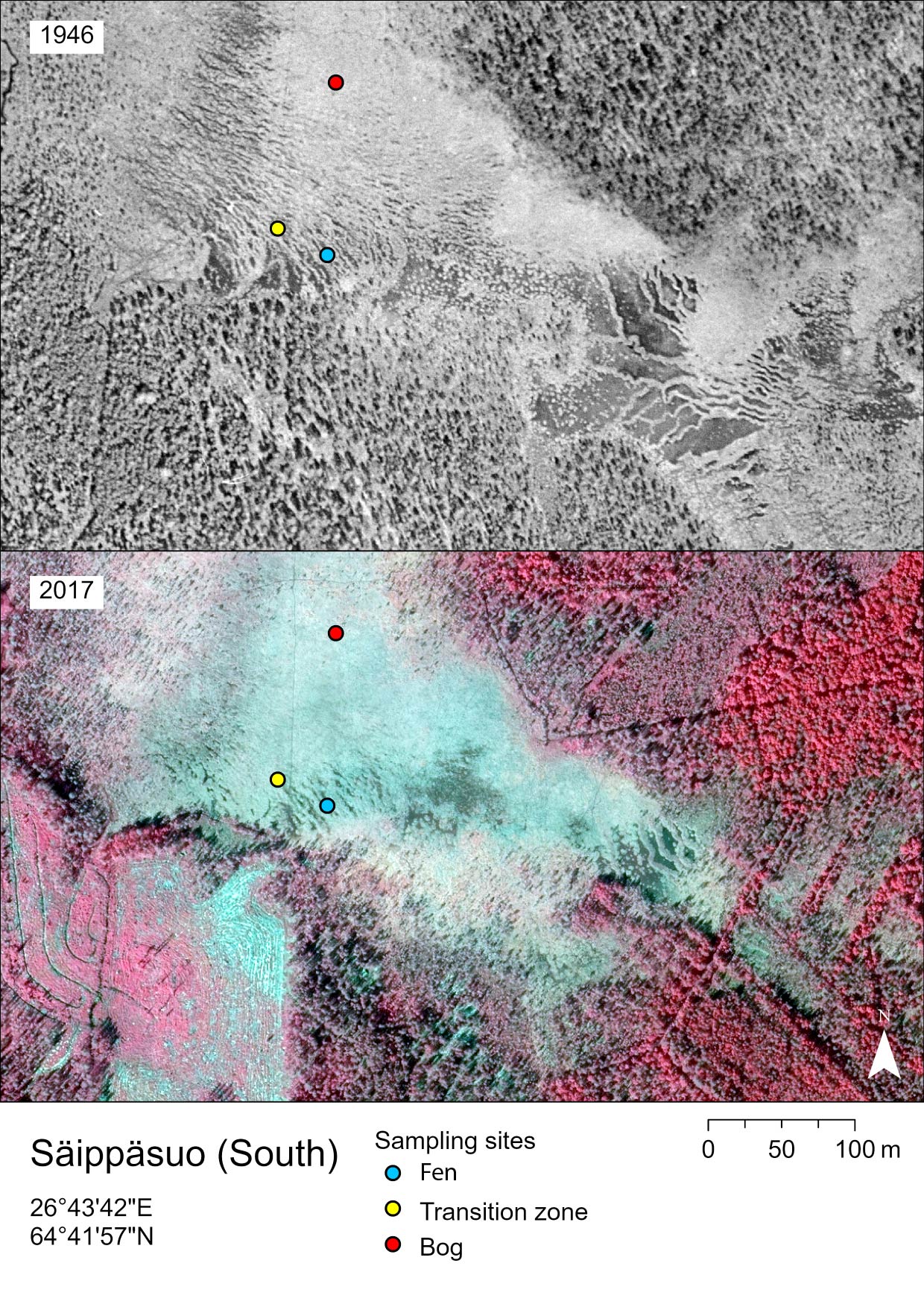


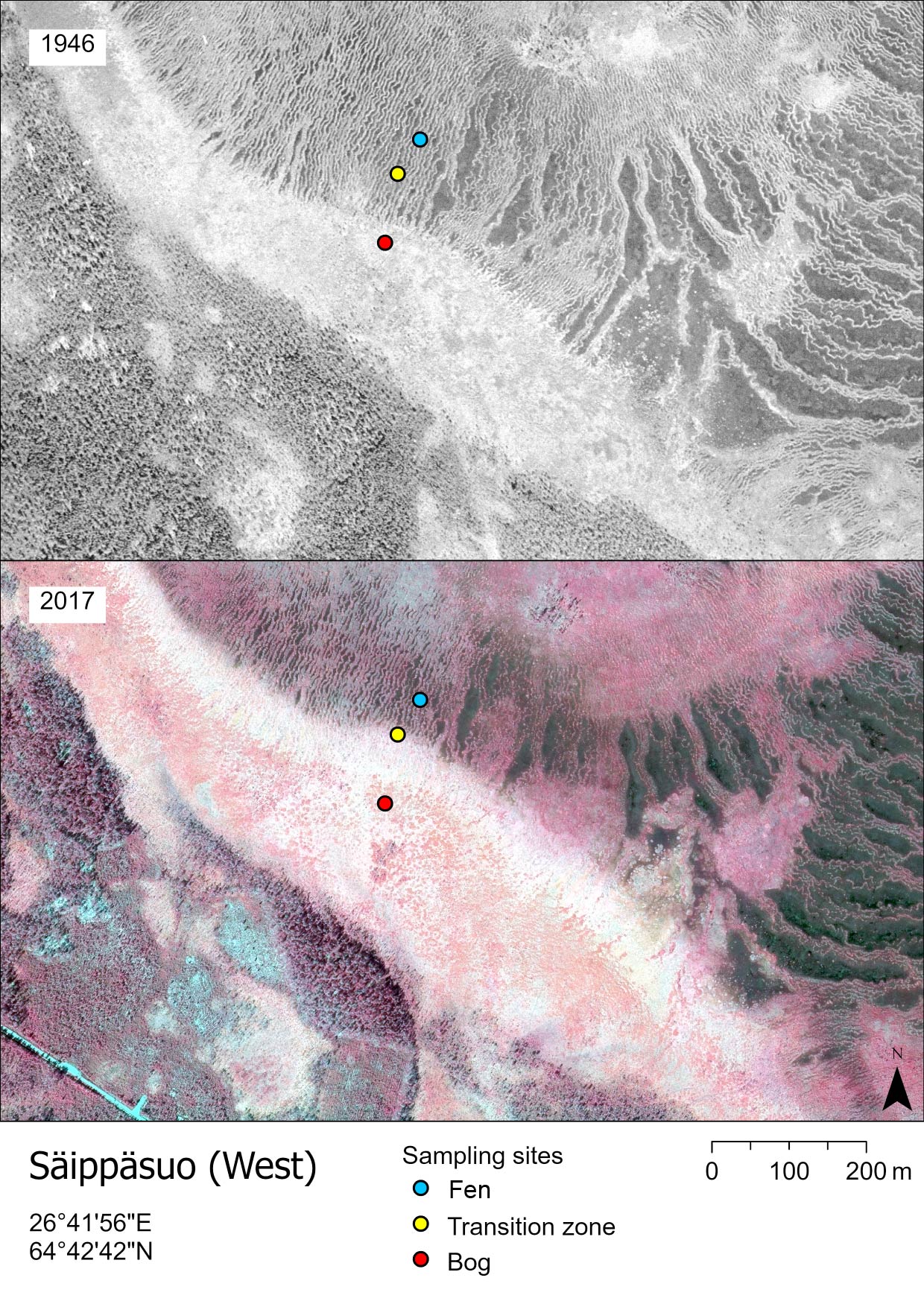


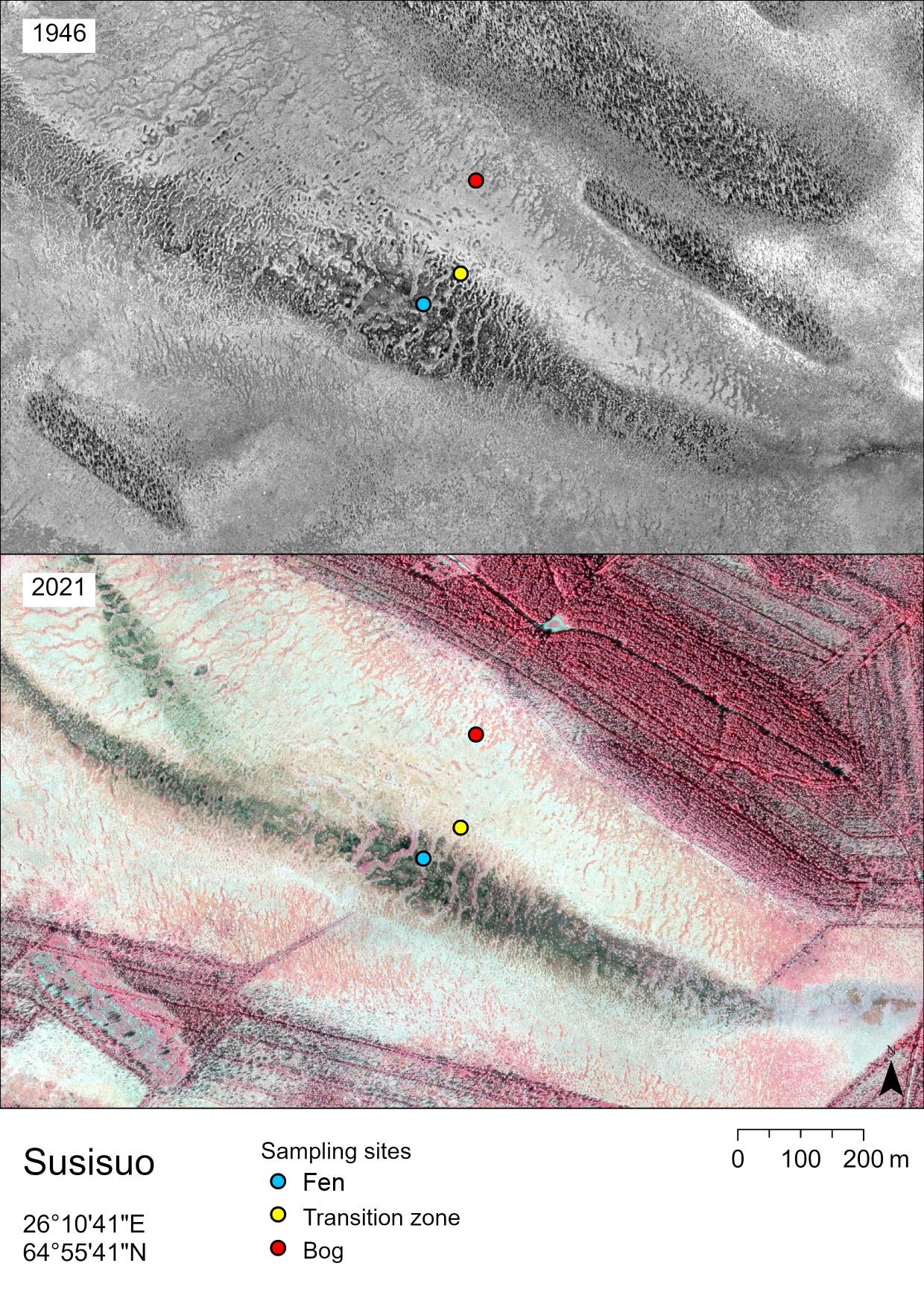


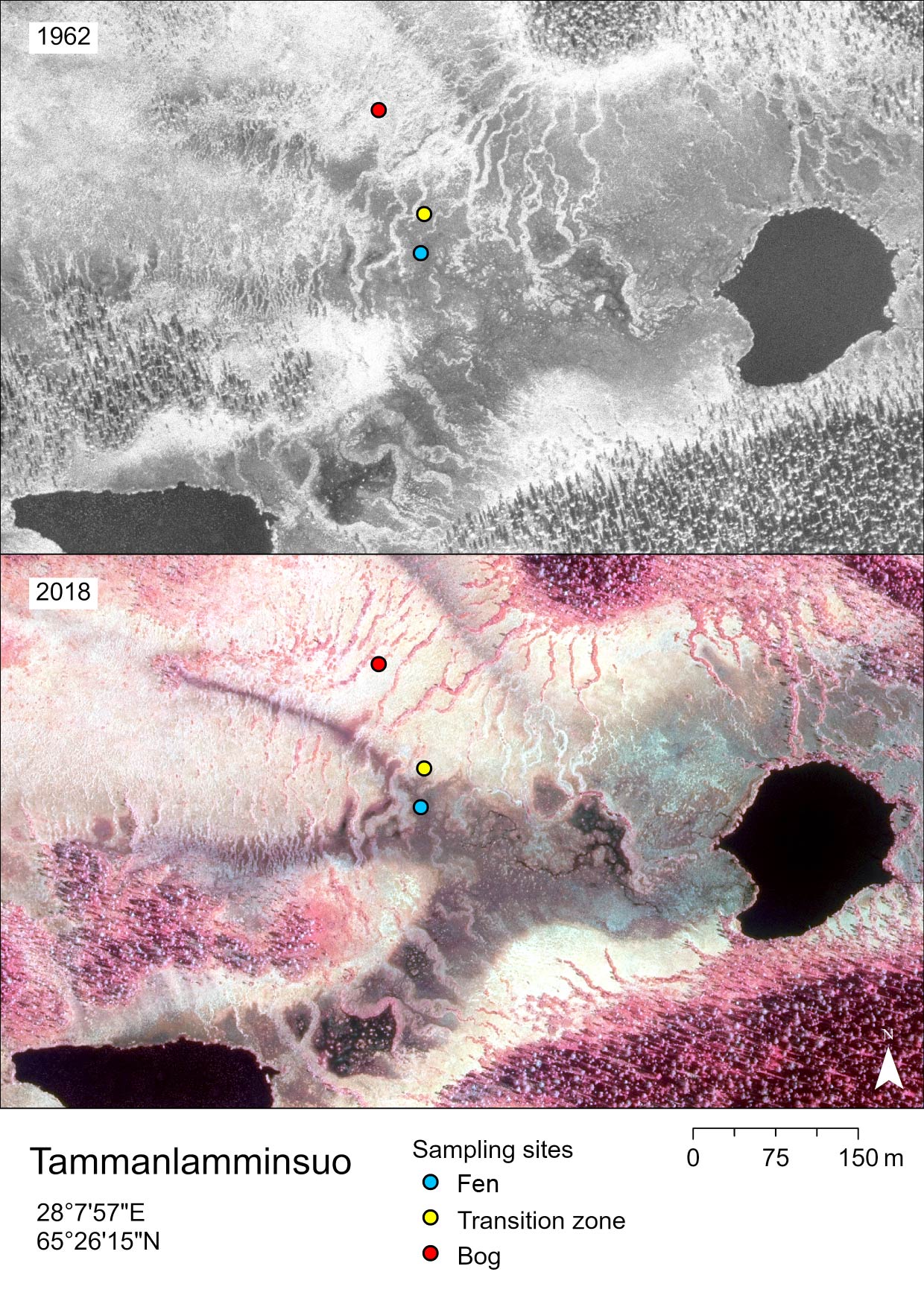


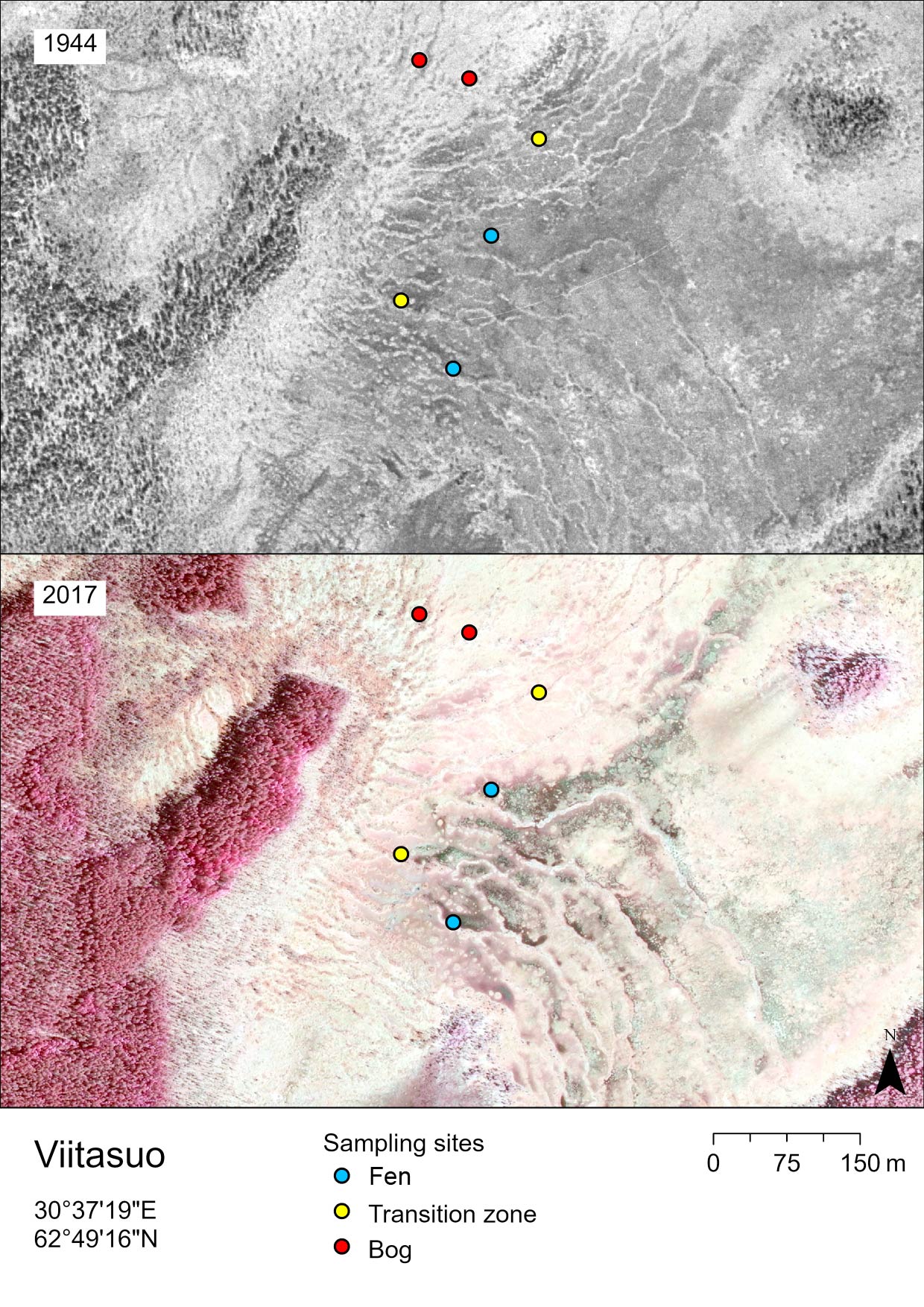

Supplement: Supplementary file 1 — Appendix S1. [file ECE3-13-e9988-s001.docx]
